# Evaluation of surveillance performance and determinants for priority vaccine-preventable diseases in Niger State, Nigeria: A CDC 2001 Guideline-based multilevel analysis

**DOI:** 10.1371/journal.pgph.0005928

**Published:** 2026-09-23

**Authors:** Oladayo David Awoyale, Akolade Jimoh, Anne Dede, Rauf Ibrahim Rauf, Arab Mustafa, Tosin Ayokunmi Sowade, Patrick B. Gimba, Idris Ibrahim, Catherine Nabiem Akpen, Eugene Chidi Eugene, Grace Fubara Erekosima, Chibuike Okekeigwe, Moses Job Tarfa, Saheed D. Isiaka, Hilary Okagbue, Sunday Atobatele, Sidney Sampson

**Affiliations:** 1 Sydani Initiative for International Development, Sydani Group, Abuja, Nigeria; 2 Sydani Institute for Research and Innovation, Sydani Group, Abuja, Nigeria; 3 Department of Statistics, Faculty of Sciences, University of Abuja, Abuja, Nigeria; 4 The World Bank, Abuja, Nigeria; 5 Ministry of Secondary and Tertiary Health, Minna, Niger State, Nigeria; University of St Andrews, UNITED KINGDOM OF GREAT BRITAIN AND NORTHERN IRELAND

## Abstract

Vaccine-preventable diseases (VPDs) remain important causes of morbidity and mortality in sub-Saharan Africa, and evidence on subnational surveillance performance in Nigeria remains limited. We conducted a cross-sectional analytic evaluation of surveillance systems for measles, diphtheria, acute flaccid paralysis (AFP), and cerebrospinal meningitis (CSM) in Niger State, Nigeria. Data were collected from 415 clinicians, community resource persons, and surveillance focal persons across 12 purposively selected Local Government Areas using structured questionnaires adapted from CDC surveillance evaluation guidelines and aligned with IDSR guidance. Surveillance performance was assessed using sensitivity, data quality, and stability. Analyses included descriptive statistics, bivariate tests, and multivariable regression models. Reported suspected case detection was higher for measles and AFP than for CSM and diphtheria, with detection reported by 94.2% and 93.0% of respondents for measles and AFP, respectively, compared with 84.3% for CSM and 81.4% for diphtheria. Transport availability without delays was consistently associated with lower odds of surveillance failure across disease-specific models, including measles sample collection (aOR = 0.12, 95% CI: 0.05–0.28) and case confirmation (aOR = 0.07, 95% CI: 0.02–0.21), diphtheria sample collection (aOR = 0.18, 95% CI: 0.06–0.49) and confirmation (aOR = 0.22, 95% CI: 0.08–0.58), and CSM sample collection (aOR = 0.18, 95% CI: 0.06–0.54) and confirmation (aOR = 0.21, 95% CI: 0.07–0.61). Active guideline use was associated with AFP sample collection (aOR = 4.12, 95% CI: 1.72–9.87). Only 66.5% of respondents reported fully accurate submitted forms, and financial support was associated with lower odds of instability (aOR = 0.74, 95% CI: 0.56–0.97). Strengthening transport, infrastructure, financing, and data quality systems may improve VPD surveillance performance and outbreak preparedness in similar settings.

## Introduction

Vaccine-preventable diseases (VPDs) remain major contributors to morbidity and mortality globally, particularly in low- and middle-income countries where recurrent outbreaks continue to strain already fragile health systems [1,2]. In 2023 alone, the World Health Organization (WHO) African Region recorded substantial outbreaks of measles, diphtheria, and cerebrospinal meningitis (CSM), with several countries experiencing resurgence associated with declining immunization coverage, delayed outbreak detection, population displacement, and weak surveillance systems [3,4]. Nigeria continues to experience a disproportionately high burden of VPD outbreaks. Between 2022 and 2024, Nigeria reported over 30,000 suspected diphtheria cases with more than 1,200 associated deaths across multiple states, while measles outbreaks persisted in several northern states despite ongoing supplementary immunization activities [3,5]. Acute flaccid paralysis (AFP) surveillance remains critical following the certification of wild poliovirus interruption in Africa because circulating vaccine-derived poliovirus outbreaks continue to occur intermittently [6]. Nigeria also lies within the African meningitis belt, where seasonal outbreaks of cerebrospinal meningitis continue to cause substantial morbidity and mortality despite expanded vaccination campaigns [2].

Disease surveillance systems are central to outbreak preparedness by enabling timely detection, confirmation, reporting, and response. In Nigeria, surveillance for priority VPDs is coordinated through the Integrated Disease Surveillance and Response (IDSR) framework, which aligns with World Health Organization regional surveillance standards [1]. Under the IDSR structure, suspected cases identified at health facilities or through community-based surveillance are reported through ward focal persons to Local Government Area (LGA) Disease Surveillance and Notification Officers (DSNOs), then transmitted to state and national surveillance authorities for collation, verification, and response coordination. Surveillance focal persons and community informants support case identification, reporting, active surveillance, and specimen movement, while laboratory confirmation pathways link health facilities with designated reference laboratories for verification of priority diseases [7]. However, weaknesses in core surveillance attributes—including sensitivity, data quality, timeliness, and system stability—continue to undermine effective surveillance performance in many settings, including Nigeria [8,9].

Nigeria has experienced recurrent outbreaks of VPDs despite long-standing implementation of IDSR [1,8,10]. Niger State, located in north-central Nigeria, has reported repeated outbreaks of measles, diphtheria, cerebrospinal meningitis, and acute flaccid paralysis, reflecting persistent challenges in disease detection, laboratory confirmation, reporting consistency, and outbreak response [2,11,12]. These outbreaks underscore the need for focused evaluation of disease surveillance performance at the sub-national level, where operational constraints are often most pronounced [9,13,14].

Several surveillance evaluations conducted in Nigeria have examined disease-specific surveillance systems, including AFP surveillance, measles case-based surveillance, meningitis surveillance, and IDSR implementation assessments [8,10,15]. Most of these studies primarily used descriptive approaches or focused on single diseases, emphasizing reporting completeness, timeliness, or outbreak trends without simultaneously evaluating multiple surveillance attributes or accounting for health system heterogeneity across facilities and LGAs. Consequently, evidence remains limited regarding how institutional, infrastructural, and operational factors jointly influence VPD surveillance performance across different diseases within routine surveillance systems.

Evidence from Nigeria highlights recurring thematic weaknesses in VPD surveillance. Evaluations conducted in Kaduna and Edo States have reported suboptimal data quality marked by incomplete or inaccurate reporting, delays in specimen transport and laboratory confirmation, and limited system stability due to resource and workforce constraints [8,10]. In conflict-affected Borno State, community informants and digital reporting tools have expanded surveillance reach and improved sensitivity; however, these gains remain uneven and fragile without sustained logistical and infrastructural support [15]. Similar logistical and infrastructural constraints observed in the present study suggest that Niger State shares broader systemic challenges documented in other Nigerian settings.

Across sub-Saharan Africa, disease surveillance evaluations reveal patterns that are directly relevant to Nigeria’s VPD surveillance context. Improvements in reporting timeliness and sensitivity are often achieved during outbreak responses; however, these gains are frequently offset by persistent weaknesses in data quality, laboratory capacity, specimen transport, and system stability once emergency support wanes [13,14,16]. These challenges mirror recurring constraints documented in Nigerian surveillance systems, where improvements in outbreak detection are often limited by incomplete reporting, delayed laboratory confirmation, inadequate logistics, and uneven sub-national implementation capacity [8,10,15].

Nigeria’s location within the African meningitis belt further reinforces the importance of robust integrated surveillance for diseases such as cerebrospinal meningitis. Across the belt, limited laboratory integration and delayed detection continue to constrain surveillance effectiveness despite the high epidemic potential and public health importance of meningitis [2]. Similarly, retrospective application of the 7–1–7 benchmark in Senegal demonstrated delays in detection and response activation, highlighting system-level bottlenecks that are also relevant for assessing sub-national surveillance performance in Nigeria [11]. Taken together, these regional findings provide a useful comparative basis for evaluating VPD surveillance performance in Niger State and for identifying operational gaps that may be shared across similar low-resource surveillance settings [2,11].

Technological and digital innovations have increasingly been applied to strengthen surveillance sensitivity, reporting, and monitoring in low-resource settings. These include mobile phone–based reporting and alert systems, electronic surveillance and data collection platforms, digital tools for specimen referral and tracking, and geospatial mapping approaches to identify underserved or hard-to-reach populations [17]. In Nigeria and similar low-resource settings, data triangulation approaches that combine surveillance, immunization, demographic, and geospatial data have also improved the identification of underserved populations and supported more targeted programmatic decision-making [18]. Nevertheless, the effectiveness and sustainability of these tools depend on enabling support functions, including workforce training, routine supervision, reliable infrastructure, logistics, and financing, which remain insufficient in many settings, including parts of Nigeria [19].

Beyond Africa, similar challenges have been documented globally. Evaluations of disease surveillance systems report delays in reporting, incomplete case information, workforce gaps, and limited epidemic preparedness in both hospital- and primary care-based surveillance systems [20,21]. These findings indicate that surveillance performance challenges are not unique to African settings, but reflect broader health system issues related to reporting structures, workforce capacity, data quality, preparedness, and the routine use of surveillance information for public health action. In South Africa and Saudi Arabia, assessments have highlighted weaknesses in timeliness, flexibility, and data use, reinforcing the need for systematic and context-sensitive surveillance evaluations [9,22].

The Centers for Disease Control and Prevention (CDC) 2001 Updated Guidelines for Evaluating Public Health Surveillance Systems provide a standardized framework for assessing key disease surveillance attributes, including sensitivity, data quality, and system stability. Despite widespread adoption of IDSR in Nigeria, available evidence remains concentrated on outbreak-specific reports, single-disease evaluations, or descriptive assessments of surveillance performance. Few studies have explicitly applied these guidelines to evaluate multiple priority VPDs simultaneously at the state level using analytical approaches that account for health system heterogeneity across facilities and local government areas.

This gap is especially evident in Niger State, where recurrent outbreaks of VPDs persist but comprehensive, guideline-based evaluations of disease surveillance performance remain scarce. In particular, limited evidence exists on how facility-level practices, workforce capacity, reporting systems, infrastructure, logistics, and financial resources jointly influence surveillance sensitivity, data quality, and stability. Understanding these relationships is critical for identifying modifiable system bottlenecks and strengthening outbreak preparedness, early detection, laboratory confirmation, reporting consistency, and response capacity at the sub-national level.

**Aim of the study:** This study evaluates the performance of disease surveillance systems for measles, diphtheria, poliomyelitis (acute flaccid paralysis), and cerebrospinal meningitis in Niger State, Nigeria, using the CDC 2001 Updated Guidelines for Surveillance System Evaluation. Specifically, it assesses the disease surveillance attributes of *sensitivity*, *data quality*, and *stability*, and examines their institutional, infrastructural, and logistic determinants.

### Conceptual framework

This study is guided by a conceptual framework adapted from the *CDC 2001 Updated Guidelines for Evaluating Public Health Surveillance Systems*. The CDC framework provides the basis for assessing whether a surveillance system is able to perform its essential public health functions, including case detection, reporting, confirmation, analysis, feedback, and response. In this study, the framework was used not only to describe surveillance performance, but also to justify the methodological choices made in the study, including the selection of surveillance attributes, the operational definition of study outcomes, the choice of explanatory variables, and the analytical strategy used to examine determinants of surveillance performance.

Within the CDC framework, surveillance performance is understood as the result of interactions between **core surveillance functions**, **support functions**, and the broader operational context in which surveillance activities are implemented. For vaccine-preventable disease (VPD) surveillance within Nigeria’s Integrated Disease Surveillance and Response (IDSR) system, these interactions are particularly important because suspected cases must be detected at facility or community level, reported through the health system, investigated, linked to laboratory confirmation where required, and acted upon through timely outbreak response. Evidence from Nigeria and other low-resource settings shows that gaps in reporting completeness, laboratory confirmation, specimen transport, supervision, workforce capacity, and logistics can weaken the performance of routine surveillance systems [1,8,10,15].

The framework therefore informed the selection of the three primary surveillance attributes assessed in this study: **sensitivity**, **data quality**, and **stability**. These attributes were prioritized because they represent operationally measurable dimensions of VPD surveillance performance and are directly linked to outbreak preparedness and response. Sensitivity reflects the extent to which the system can detect suspected cases, support sample collection, and facilitate laboratory confirmation. Data quality reflects the completeness, consistency, and accuracy of surveillance information generated through routine reporting processes. Stability reflects the ability of the system to function continuously despite disruptions in staffing, logistics, infrastructure, financing, and commodity availability.

The selection of these attributes is also supported by surveillance evaluation evidence from Nigeria and wider sub-Saharan African settings. Studies from Nigeria have reported recurring challenges related to incomplete reporting, delayed laboratory confirmation, weak logistics, and uneven surveillance implementation capacity [8,10,15]. Similar weaknesses have been documented in other African settings, where surveillance performance has been constrained by delayed case detection, incomplete data, limited laboratory integration, weak feedback systems, workforce shortages, and unstable funding or logistics support [2,9,13,14,16]. These findings demonstrate that the selected attributes are not arbitrary, but reflect recurring surveillance system weaknesses observed across comparable low-resource and outbreak-prone settings.

**Core surveillance functions** in the framework include case detection and registration, specimen collection, laboratory confirmation, routine reporting, data analysis and interpretation, and feedback mechanisms. These functions determine whether suspected VPD cases are identified, verified, documented, and communicated across health system levels for timely public health action [1,8]. In the present study, these functions informed the measurement of sensitivity through indicators such as reported suspected case detection, sample collection, laboratory-confirmed cases, and case misclassification.

**Support functions** include workforce training and supervision, availability and use of surveillance guidelines, logistics and specimen transportation systems, financing, infrastructure, electricity supply, refrigerator functionality, digital reporting systems, and coordination mechanisms [19,23]. These support functions were included as explanatory variables because they are expected to influence how well core surveillance functions are performed. For example, transport availability may affect sample movement and laboratory confirmation; power supply and refrigerator functionality may affect specimen integrity; training and guideline use may affect correct case identification; and financing may affect continuity of surveillance operations.

This conceptual framework directly informed the study methodology in four ways. First, it guided the selection of study outcomes by identifying sensitivity, data quality, and stability as priority CDC-aligned surveillance attributes. Second, it guided the operationalization of these outcomes into measurable indicators, including any sample collection, any laboratory-confirmed case, ≥80% and ≥90% data completeness/accuracy, and stability thresholds based on operational continuity indicators. Third, it guided the selection of independent variables by linking facility-level practices, workforce characteristics, infrastructure, logistics, financing, and guideline use to expected surveillance performance. Fourth, it justified the use of multivariable and multilevel regression models, since surveillance performance is influenced by factors operating at respondent, facility, ward, and LGA levels.

**Fig 1:**
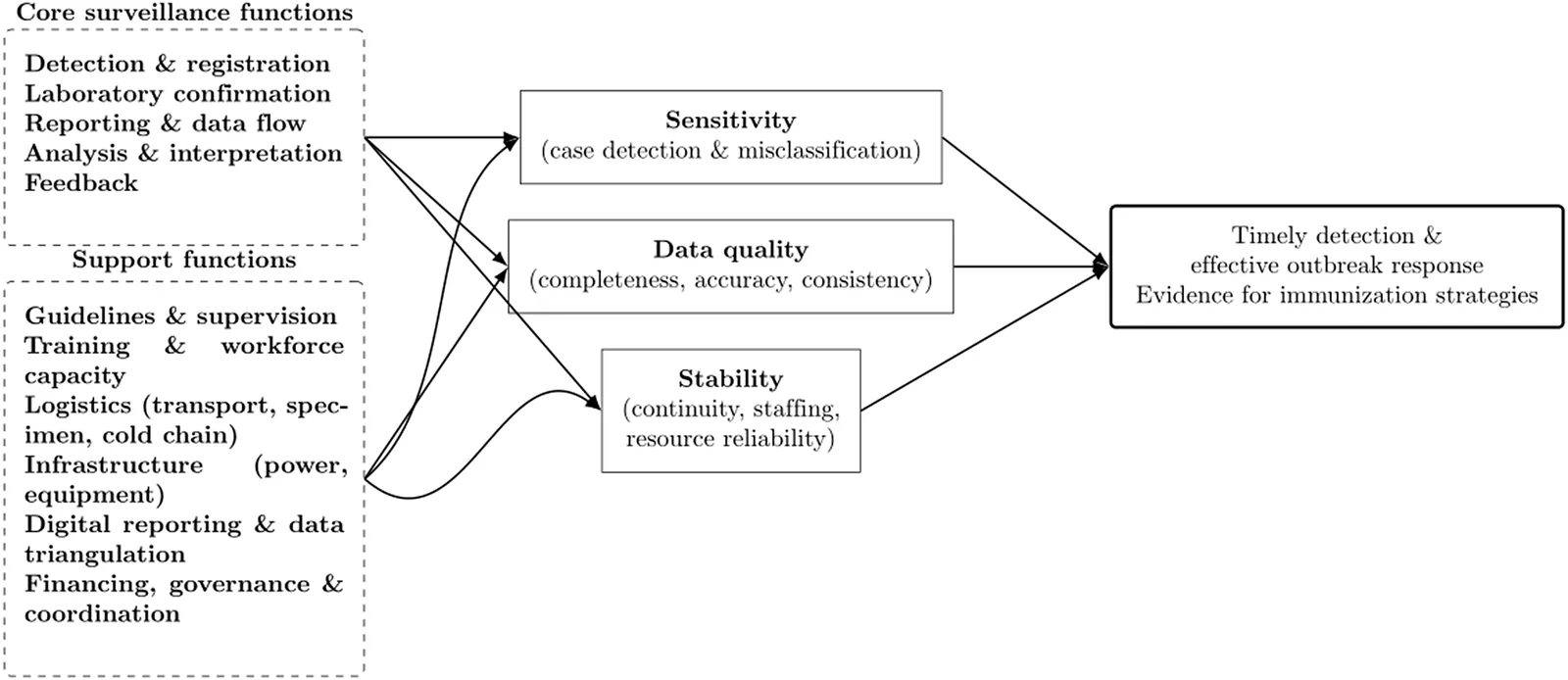
Conceptual framework linking IDSR core and support functions to surveillance attributes (sensitivity, data quality, stability) and downstream outcomes for vaccine-preventable disease (VPD) control.

In this framework, surveillance attributes are treated as intermediate operational outcomes through which core and support functions influence broader outbreak preparedness and response capacity. The framework assumes that weaknesses in logistics, financing, supervision, transport systems, workforce capacity, infrastructure reliability, and guideline utilization may reduce surveillance sensitivity, compromise data quality, and weaken system stability. These weaknesses may subsequently undermine timely outbreak detection, laboratory confirmation, reporting consistency, and effective public health response. Thus, the conceptual framework Fig 1 provides the methodological foundation for evaluating not only whether the VPD surveillance system is performing adequately, but also which modifiable operational factors are associated with better or poorer surveillance performance in Niger State.

## Methods

### Ethics statement

Ethical approval for the study was obtained from the Niger State Ministry of Health Ethical Review Committee (ERC PIN/2025/06/46) on 16/06/2025, before the commencement of data collection. Written informed consent was obtained from all participants prior to their participation in the study. Participation was voluntary, and respondents were informed that they could decline participation or withdraw from the study at any time without any consequence. No personal identifiers were included in the analysis or reporting. All data were anonymised and stored securely in accordance with institutional data protection requirements, and access was restricted to authorized members of the research team.

### Study design

We conducted a cross-sectional analytic evaluation of vaccine-preventable disease (VPD) surveillance systems in Niger State, Nigeria. The study assessed surveillance performance for measles, diphtheria, cerebrospinal meningitis (CSM), and acute flaccid paralysis (AFP) using an interviewer-administered structured surveillance evaluation tool. The tool was developed by adapting relevant domains from the *CDC 2001 Updated Guidelines for Evaluating Public Health Surveillance Systems* and the WHO *Integrated Disease Surveillance and Response* (IDSR) technical guidance. The adaptation ensured that the tool captured both standard surveillance evaluation attributes and context-specific operational realities of VPD surveillance within the Nigerian IDSR system.

The study tool contained sections on respondent socio-demographic characteristics, disease-specific surveillance functions, case detection, sample collection, laboratory confirmation, reporting practices, data quality, availability and use of surveillance guidelines, logistics and transport systems, supervision, infrastructure, financing, and system continuity. These domains were used to generate the main analytical outcomes of the study, namely surveillance sensitivity, data quality, and stability. Sensitivity was assessed using disease-specific indicators such as suspected case detection, sample collection, laboratory confirmation, and reported case misclassification. Data quality was assessed using reported completeness, consistency, and accuracy of surveillance forms and routine documentation practices. Stability was assessed using indicators of uninterrupted surveillance operations, staffing continuity, availability of forms and commodities, transport functionality, infrastructure reliability, power supply, financial support, and sustainability planning.

The tool was designed specifically for this study but was based on internationally recognized surveillance evaluation frameworks. It was reviewed for content validity by public health surveillance and epidemiology experts to ensure alignment with CDC and IDSR surveillance evaluation domains. It was also pretested before data collection to assess clarity, relevance, flow, and suitability for field administration. Feedback from the pretest was used to refine wording, response options, and sequencing of questions before final deployment.

A cross-sectional analytic design was considered appropriate because the study sought to assess surveillance attributes and their determinants across multiple system levels at a defined point in time. This design enabled comparison of surveillance performance across respondents, facilities, wards, and Local Government Areas (LGAs), while also allowing the study to examine how workforce, logistics, infrastructure, financing, reporting practices, and guideline use were associated with key surveillance outcomes. Recruitment and primary data collection were conducted from 01/07/2025 to 30/09/2025, following receipt of ethical approval on 16/06/2025.

### Study setting

Niger State is located in north-central Nigeria and comprises 25 Local Government Areas (LGAs) distributed across three senatorial districts. Disease surveillance is implemented through the Integrated Disease Surveillance and Response (IDSR) system, with routine reporting and coordination occurring across ward, LGA, and state levels within both public and private health facilities.

Twelve LGAs were purposively selected to capture epidemiological, geographic, and operational heterogeneity within the state surveillance system. Selection criteria included recurrent occurrence and reporting burden of priority vaccine-preventable diseases (VPDs), representation across the three senatorial districts, variation in urban-rural settings, and feasibility of sustained field access in the context of prevailing security conditions. Four LGAs were selected from each senatorial district to ensure broad geographic distribution and inclusion of diverse surveillance contexts within the state.

**Fig 2:**
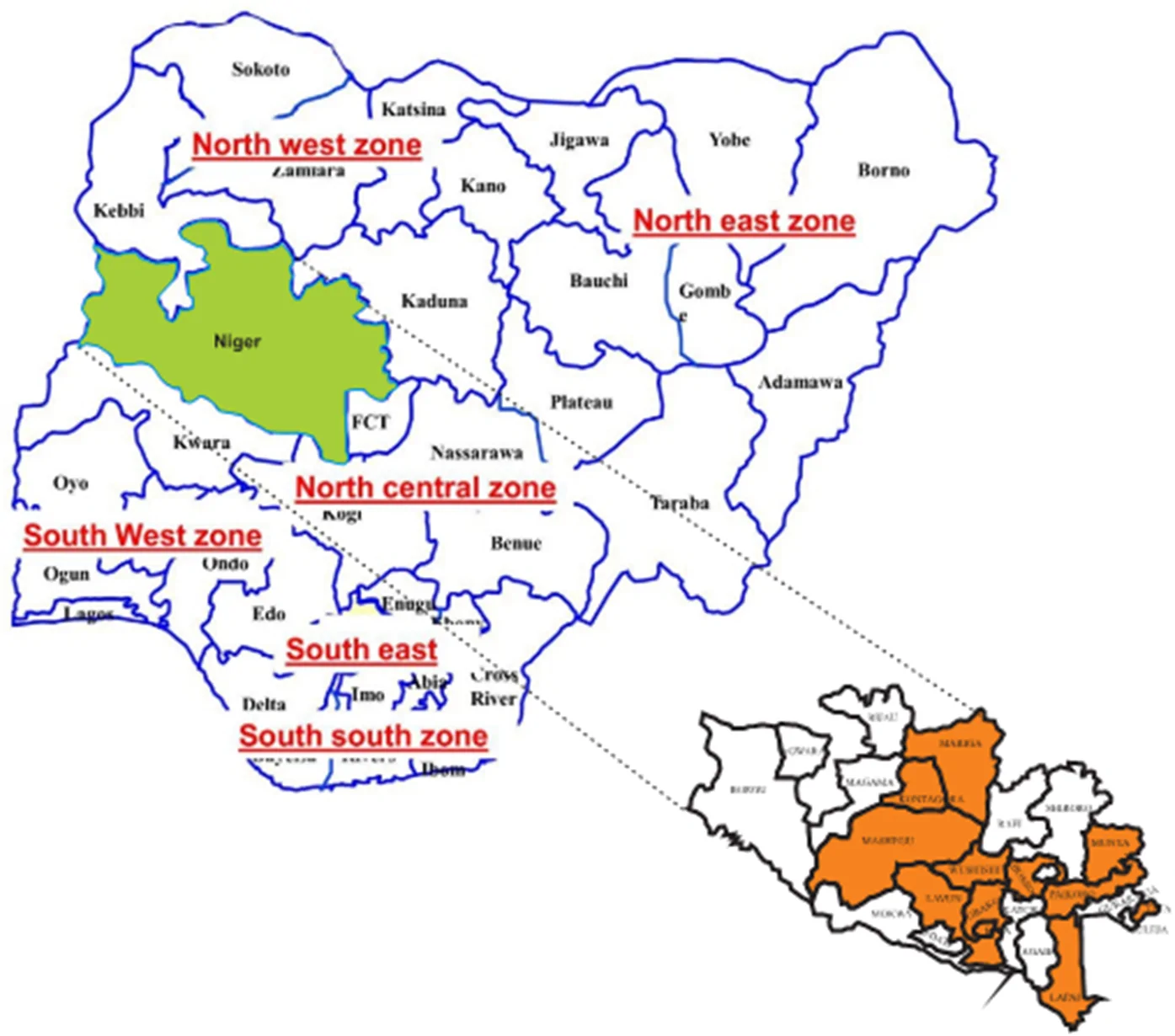
Map of Nigeria showing Niger State. Administrative boundaries were obtained from the HDX/OCHA Nigeria Subnational Administrative Boundaries dataset (https://data.humdata.org/dataset/cod-ab-nga), licensed under CC-BY 4.0. Niger State was highlighted by the authors.

**Fig 3:**
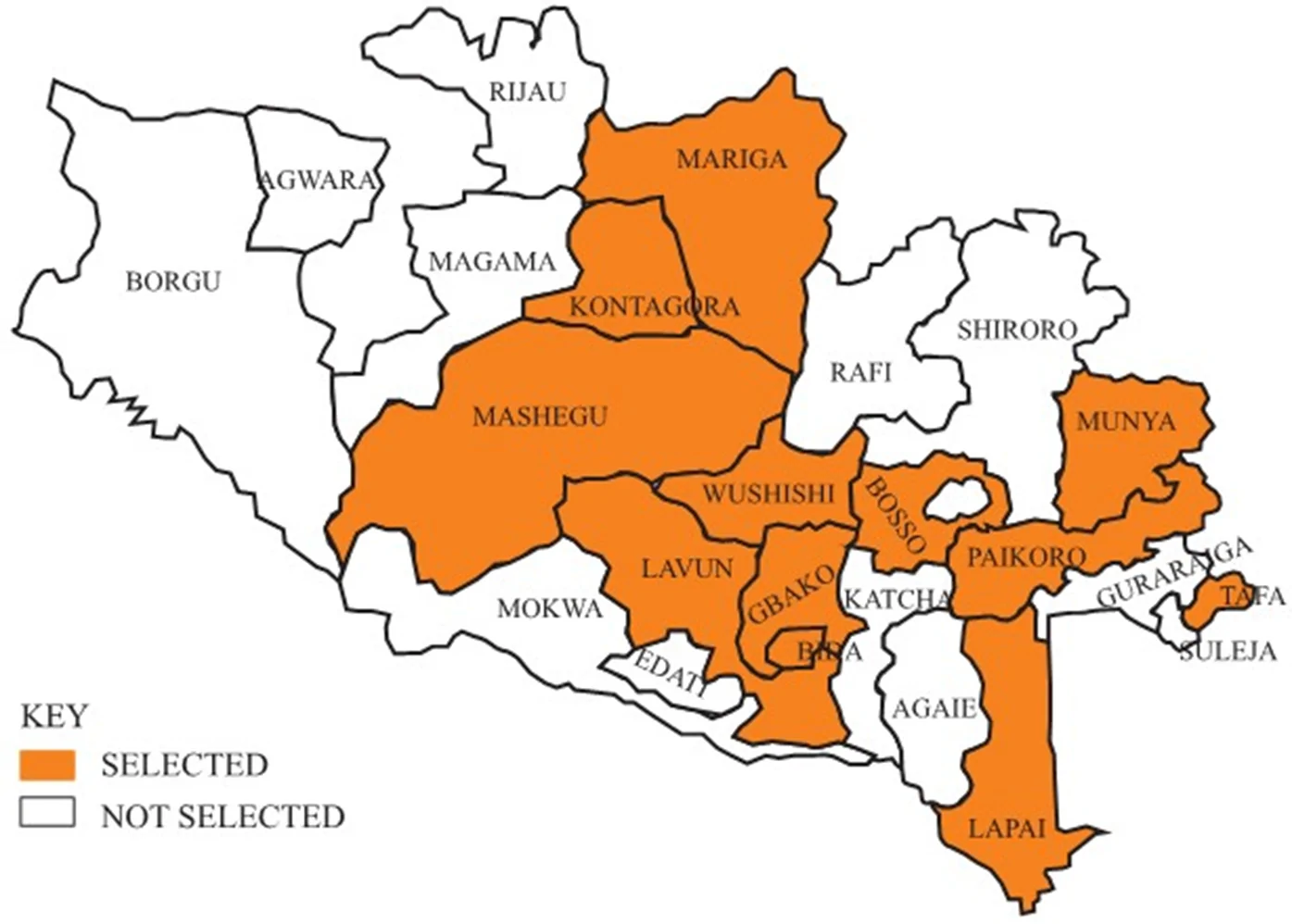
Map of Niger State showing selected Local Government Areas. Administrative boundaries were obtained from the HDX/OCHA Nigeria Subnational Administrative Boundaries dataset (https://data.humdata.org/dataset/cod-ab-nga), licensed under CC-BY 4.0. Selected LGAs were highlighted by the authors.

The purposive approach was considered appropriate for surveillance system evaluation because the study aimed to assess functionality and operational determinants of the IDSR system across settings with varying surveillance demands and implementation realities, rather than to generate population-based prevalence estimates. Inclusion of LGAs with differing epidemiological profiles, infrastructure conditions, and accessibility constraints allowed assessment of surveillance performance under routine operational conditions commonly encountered within the broader Niger State health system. Figs 2 and 3 present the national and state-level study context, respectively.

### Study population

The study population comprised frontline personnel directly involved in vaccine-preventable disease (VPD) surveillance at ward and Local Government Area (LGA) levels in Niger State. These included Surveillance Focal Persons (SFPs), Community Resource Persons (CRPs), and clinicians who participated in case detection, notification, reporting, sample referral, laboratory follow-up, supervision, or other surveillance-related activities for measles, diphtheria, cerebrospinal meningitis (CSM), and acute flaccid paralysis (AFP).

Eligible participants were personnel who were actively involved in VPD surveillance activities within the selected wards and LGAs during the study period, had worked in a surveillance-related role for a sufficient period to provide information on routine surveillance operations, were available during data collection, and provided informed consent to participate in the study. Personnel who were not involved in VPD surveillance activities, were newly posted and unable to provide information on surveillance processes, or did not provide consent were excluded.

### Sample selection and allocation

A purposive sampling approach was used to select eligible surveillance personnel from the selected LGAs and wards. This approach was considered appropriate because the target respondents were not members of the general population but a defined group of health workers and community-level surveillance actors with direct knowledge of VPD surveillance operations. Therefore, respondents were selected based on their involvement in surveillance activities and their ability to provide relevant information on surveillance sensitivity, data quality, stability, reporting practices, logistics, and system functionality.

The selected LGAs were distributed across the three senatorial districts of Niger State to reflect geographic and operational variation in the state surveillance system. Within each selected LGA, the sample was allocated proportionally according to the number of wards. This ensured that LGAs with more wards contributed a larger number of respondents while maintaining representation across all selected LGAs. Within each ward, eligible surveillance personnel were identified in collaboration with relevant LGA and ward-level surveillance structures. Personnel who met the inclusion criteria and provided informed consent were recruited until the allocated sample for each LGA was achieved. The final sample consisted of 415 consenting surveillance personnel across the 12 selected LGAs (Table 1).

**Table 1:** Sample allocation across selected Local Government Areas (LGAs) in Niger State.

LGA	Bida	Bosso	Gbako	Kontagora	Lapai	Lavun	Mariga	Mashegu	Munya	Paikoro	Tafa	Wushishi	Total
**Number of wards**	14	10	10	13	10	12	11	10	11	11	10	11	**133**
**Allocated and consenting sample**	43	31	31	40	31	37	34	31	34	34	31	33	**415**

Notes: LGAs were selected across the three senatorial districts of Niger State. Sample allocation was proportional to the number of wards in each selected LGA. Within each ward, eligible frontline surveillance personnel who met the inclusion criteria and provided informed consent were recruited until the allocated LGA sample was achieved.

### Sampling procedure

A multistage sampling approach was used (Table 2). First, Niger State was stratified into its three senatorial districts to ensure geographic representation across the state and to capture potential variation in surveillance system performance, infrastructure, and operational context. Within each district, four Local Government Areas (LGAs) were purposively selected based on epidemiological risk profiles, history of vaccine-preventable disease outbreaks, functionality of surveillance activities, and security accessibility. Purposive selection was considered appropriate for this surveillance system evaluation because the study aimed to assess surveillance performance in operationally relevant and high-priority settings where vaccine-preventable disease detection and response activities are most actively implemented. This approach also ensured inclusion of LGAs with varying surveillance realities across the state while maintaining feasibility of field implementation.

**Table 2:** Multistage sampling approach used in the study.

Stage	Sampling technique	Description
Stage 1	Stratification	Niger State was stratified into the three senatorial districts to ensure geographic spread and representation of the major administrative zones of the state.
Stage 2	Purposive selection of LGAs	Four LGAs were selected from each senatorial district based on epidemiological relevance, history or risk of VPD outbreaks, operational feasibility, and security accessibility. This resulted in 12 selected LGAs.
Stage 3	Inclusion of wards within selected LGAs	All wards within the selected LGAs were included as operational sampling units. This ensured that respondent selection was spread across the ward surveillance structure rather than concentrated in a few locations.
Stage 4	Purposive selection of eligible surveillance personnel	Within the selected wards and LGAs, eligible surveillance personnel were identified across the main surveillance-related cadres, including Surveillance Focal Persons (SFPs), Community Resource Persons (CRPs), and clinicians. Respondents were selected based on active involvement in VPD surveillance activities, availability during data collection, relevant experience, and willingness to provide informed consent. Selection was not based on a fixed assumption of one facility or one respondent per cadre in every ward, because ward-level staffing and facility distribution varied across LGAs. Recruitment continued until the allocated sample for each LGA was achieved.

Notes: Sampling emphasized epidemiological relevance, operational feasibility, and geographic coverage across the three senatorial districts.

All wards within selected LGAs were included to maximize coverage of ward-level surveillance structures and reporting processes. Surveillance personnel were subsequently stratified by cadre, including Surveillance Focal Persons (SFPs), Community Resource Persons (CRPs), and clinicians. One eligible respondent per cadre per ward was purposively selected based on active involvement in surveillance activities and minimum required experience in the current role.

### Eligibility criteria

Participants were eligible for inclusion if they were actively involved in vaccine-preventable disease (VPD) surveillance activities within the selected wards or LGAs during the study period. Eligible respondents included Surveillance Focal Persons (SFPs), Community Resource Persons (CRPs), and clinicians who participated in case detection, notification, reporting, sample referral, laboratory follow-up, supervision, or other surveillance-related activities for measles, diphtheria, cerebrospinal meningitis (CSM), and acute flaccid paralysis (AFP).

To ensure that respondents had sufficient knowledge of routine surveillance processes, participants were required to have at least six months of continuous experience in their current surveillance-related role. Respondents were excluded if they were on leave, retired, transferred out of the selected ward or LGA at the time of data collection, not directly involved in VPD surveillance activities, or unable or unwilling to provide informed consent (Table 3).

**Table 3:** Eligibility criteria for study participants.

Category	Criteria
Inclusion criteria	Active Surveillance Focal Person (SFP), Community Resource Person (CRP), or clinician; at least six months of continuous experience in the current role; direct involvement in VPD surveillance activities, including case detection, reporting, sample referral, laboratory follow-up, supervision, or routine surveillance documentation.
Exclusion criteria	Personnel not directly involved in VPD surveillance activities; newly posted personnel with less than six months of experience in the current role; personnel on leave, retired, or transferred at the time of data collection; refusal or inability to provide informed consent.

**Notes:** The minimum six-month experience requirement was applied to ensure adequate familiarity with IDSR reporting procedures and routine surveillance operations.

### Data collection and management

Data were collected using a structured questionnaire adapted from the **CDC 2001 Updated Guidelines** and WHO IDSR tools. Questionnaires were administered in person to eligible surveillance personnel. Most variables, including surveillance practices, availability of resources, interruptions, reporting processes, and perceived system functionality, were based on respondent self-report rather than independent facility audits. To reduce recall bias, questions were framed around defined and recent reference periods where applicable, such as the preceding month or the preceding six months. To minimize social desirability bias, trained data collectors used standardized wording, assured respondents that their responses would be anonymised and used only for research purposes, and emphasized that participation would not affect their employment or relationship with the health system.

Completed questionnaires were reviewed for completeness before data entry. Data were entered in Microsoft Excel 365 and analysed using STATA version 15. Data cleaning included duplicate removal, range and consistency checks, review of implausible values, and assessment of missing values. Cleaned datasets were anonymised and stored on password-protected devices with restricted access.

### Descriptive analysis

Participant characteristics and surveillance system features were summarised using frequencies and percentages for categorical variables and means (SD) or medians (IQR) for continuous variables, as appropriate. Analyses were conducted in Microsoft Excel 365 and STATA 15.

### Surveillance performance evaluation

Surveillance performance for each vaccine-preventable disease (VPD) was evaluated using selected attributes adapted from the **CDC 2001 Updated Guidelines for Evaluating Public Health Surveillance Systems** and aligned with WHO Integrated Disease Surveillance and Response (IDSR) guidance. The evaluation focused on three key attributes: **sensitivity**, **data quality**, and **stability**, as these reflect core operational dimensions of surveillance performance in outbreak-prone and resource-constrained settings.

**Sensitivity** was defined as the ability of the surveillance system to detect, report, investigate, and support laboratory confirmation of suspected VPD cases. It was assessed using indicators such as suspected case detection, new case identification, specimen collection, laboratory confirmation, and reported case misclassification. Disease-specific indicators included non-polio AFP detection and stool specimen adequacy for acute flaccid paralysis (AFP); suspected case investigation, specimen collection, and timeliness of reporting and response for measles; reporting completeness, timeliness, and lumbar puncture practices for cerebrospinal meningitis (CSM); and suspected case reporting, laboratory confirmation, and contact tracing for diphtheria.

**Data quality** referred to the completeness, accuracy, consistency, and reliability of surveillance records. It was assessed using form completeness, reported accuracy of submitted records, documentation practices, data review mechanisms, and linkage of laboratory results to surveillance reports.

**Stability** was defined as the ability of the surveillance system to function reliably over time despite operational disruptions. It was assessed using indicators related to continuity of surveillance activities, staffing, reporting material stockouts, transport reliability, infrastructure functionality, guideline availability and use, financial support, and sustainability planning.

Indicators were assessed at facility and LGA levels to capture variation in routine surveillance functionality and outbreak detection capacity within the IDSR framework.

### Inferential analysis

Associations were examined using Chi-square or Fisher’s exact tests, depending on the expected cell frequencies in the cross-tabulations. Multilevel logistic regression models with random intercepts for LGAs were fitted to identify determinants of surveillance performance while accounting for clustering. Covariates were selected based on the conceptual framework and included training status, years of experience, cadre, access to transport or diagnostics, and supervision. Statistical significance was assessed at α=0.05.

## Results

### Respondents’ socio-demographics

**Fig 4:**
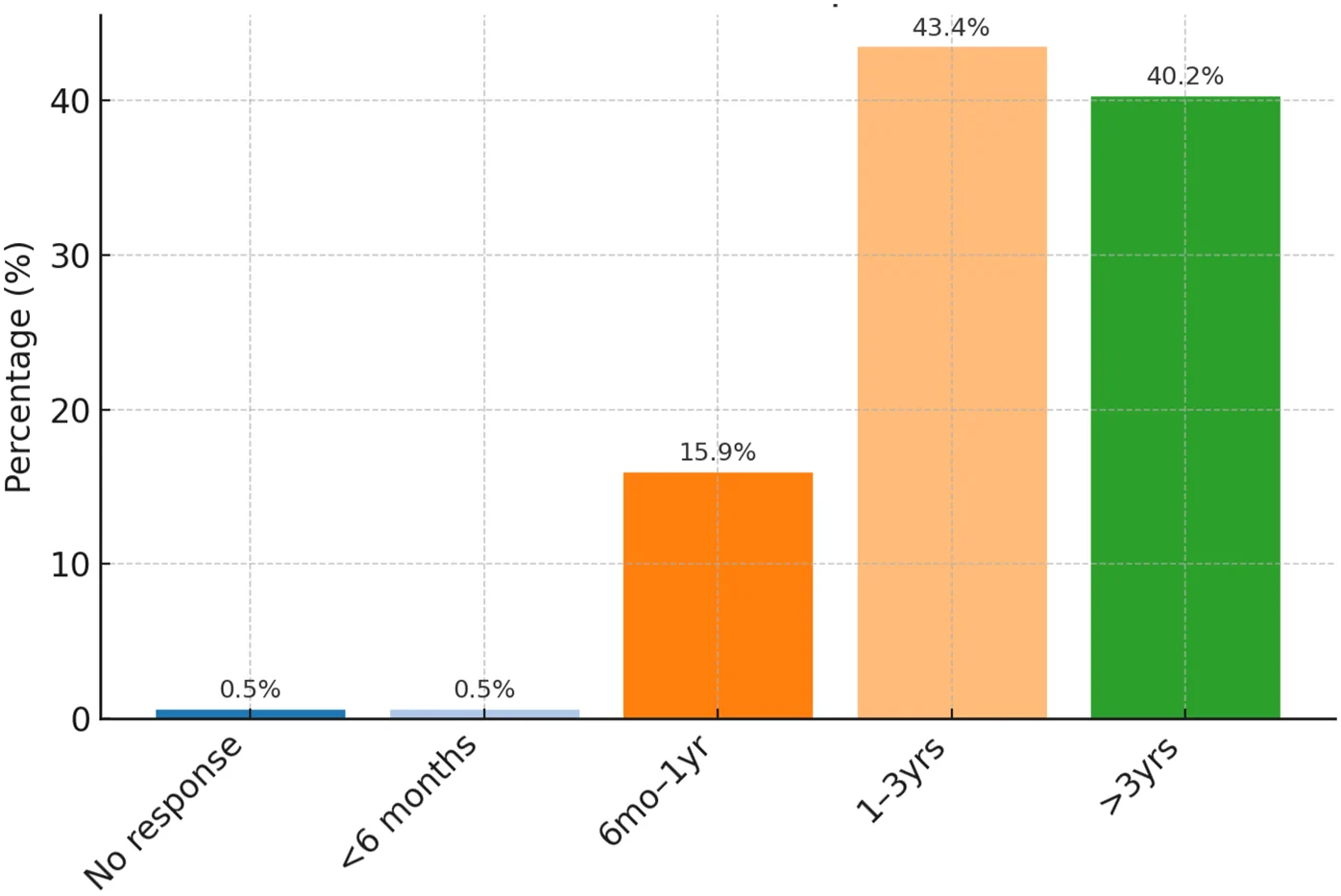
Years in role.

**Fig 5:**
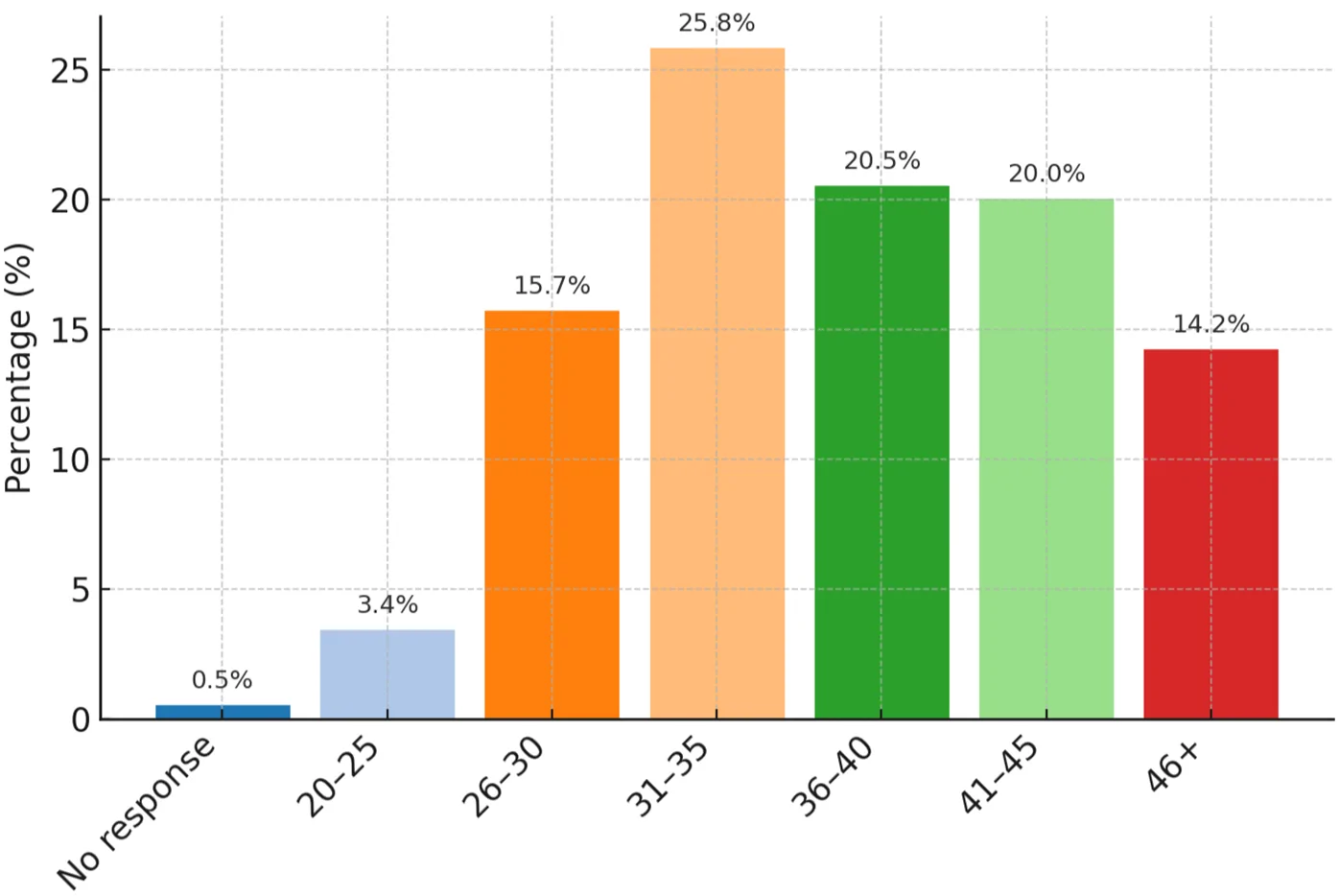
Age distribution.

A total of 415 respondents participated in the study across the 12 selected LGAs of Niger State. Records with missing socio-demographic information were excluded from percentage calculations for the affected variables (Figs 4 and 5).

**Fig 6:**
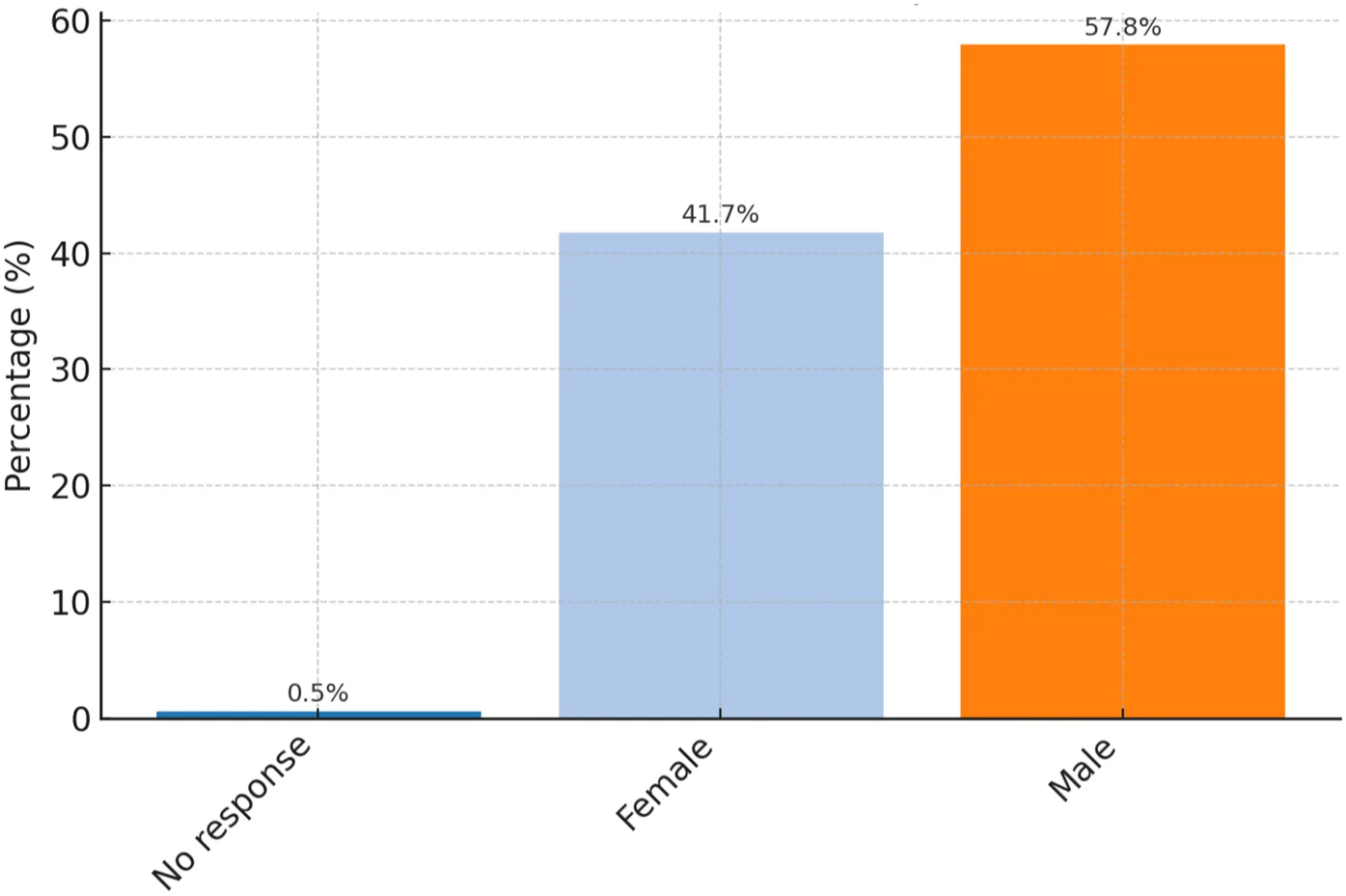
Gender distribution.

**Fig 7:**
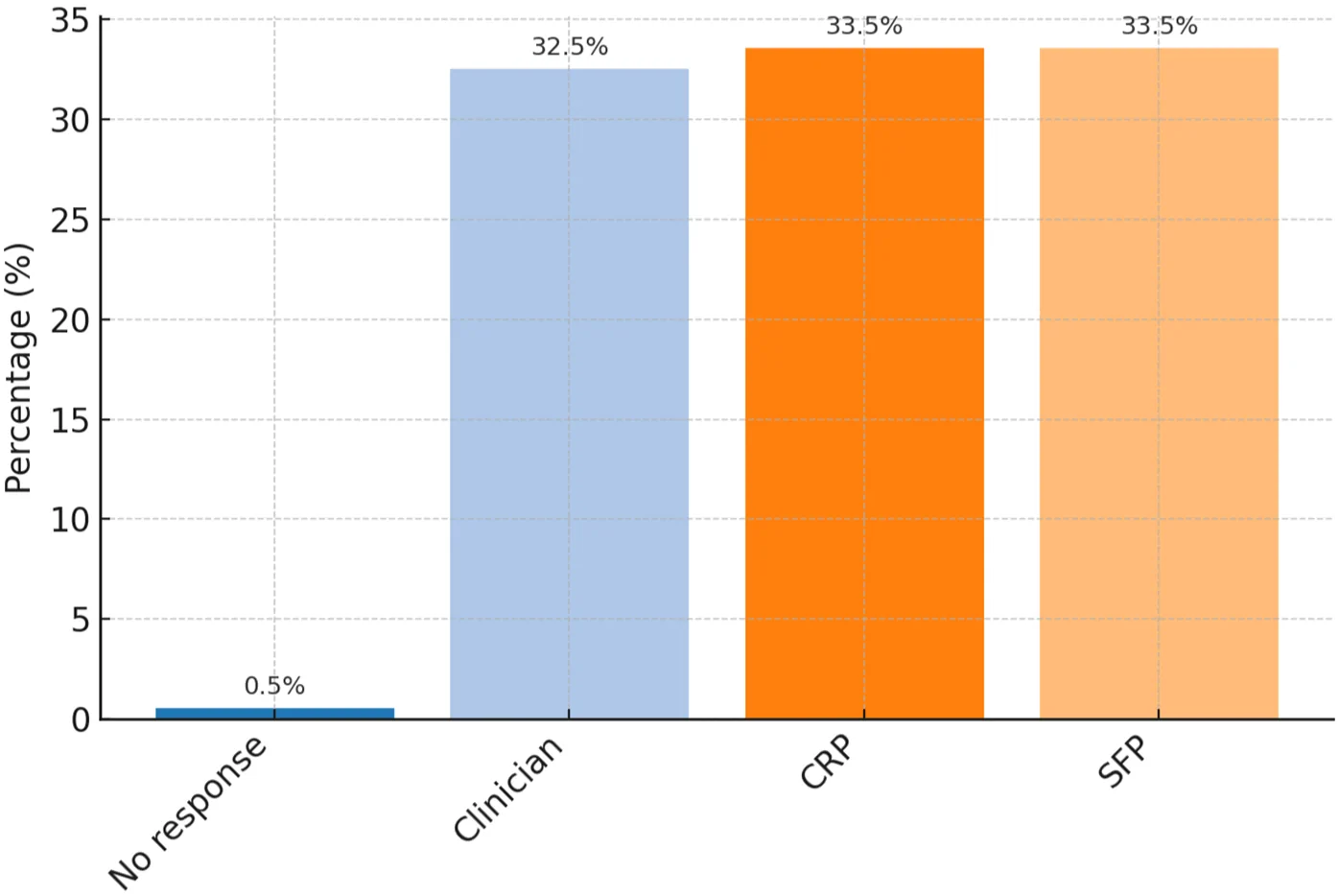
Designation.

**Fig 8:**
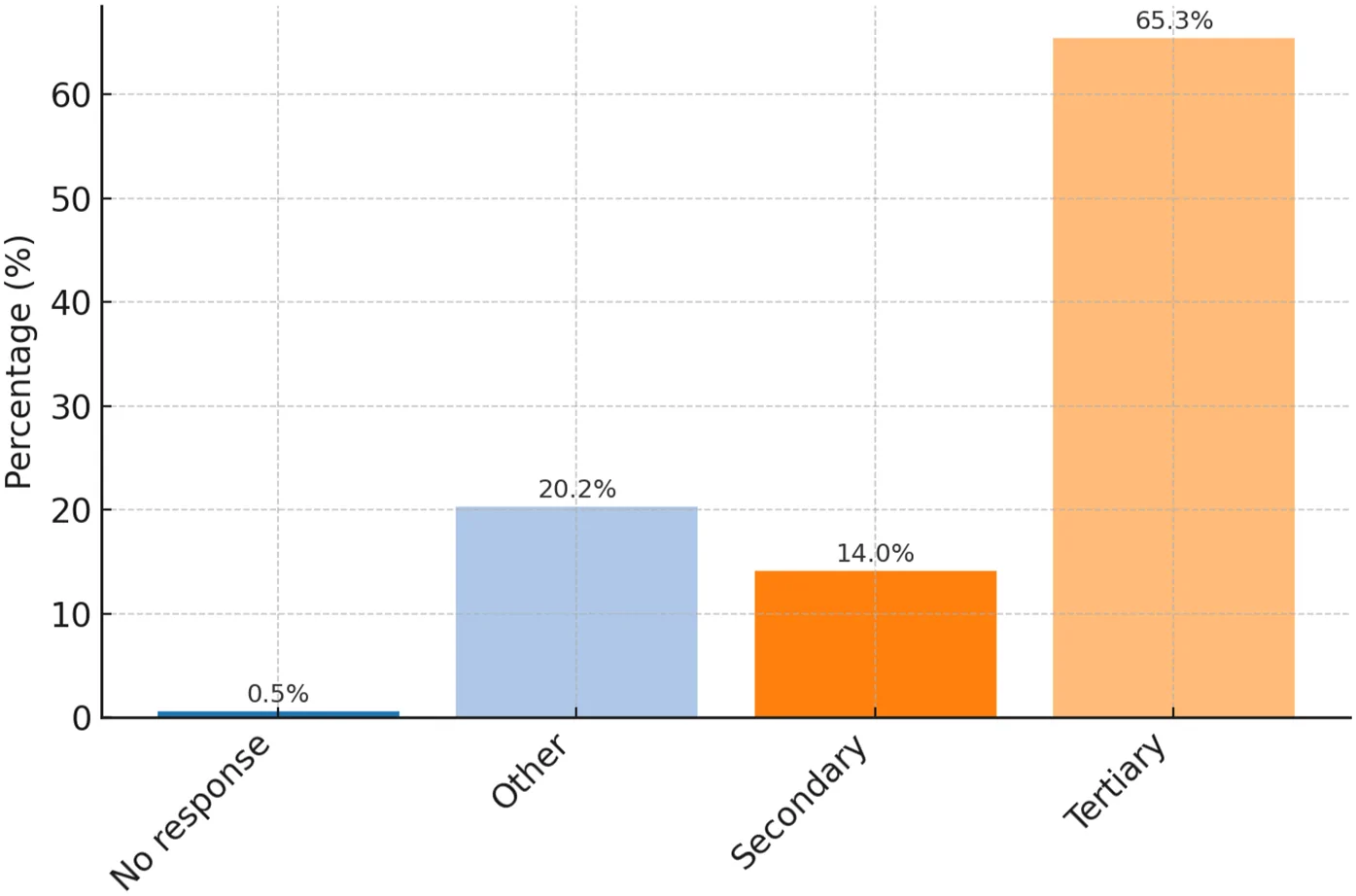
Education level.

Among respondents with complete gender data, males accounted for 57.8% while females represented 42.2% (Fig 6). The majority of respondents were aged 31–40 years (46.3%) (Fig 5). Respondents were relatively evenly distributed across the three principal surveillance cadres, with clinicians comprising 32.5%, Community Resource Persons (CRPs) 33.5%, and Surveillance Focal Persons (SFPs) 33.5% (Fig 7). Most respondents reported tertiary-level education (65.3%) (Fig 8).

With respect to work experience, most respondents had served in their current surveillance-related roles for 1–3 years (43.4%), while 40.2% reported more than three years of experience in their roles (Fig 4). The respondent population reflected a moderately experienced surveillance workforce composed primarily of formally trained personnel across clinical, community-based, and surveillance coordination roles.

### Core and support function performance

#### Sensitivity.

Sensitivity was assessed using respondents’ reports on system functionality, satisfaction with case definitions, and the frequency of reported case misclassification. These indicators describe the ability of the surveillance system to identify and classify suspected cases across the four priority vaccine-preventable diseases (VPDs).

**Perceptions of system functionality.** Most respondents reported that the surveillance system was able to detect suspected cases across all VPDs. Detection of suspected measles cases was reported by 94.2% of respondents, followed by AFP (93.0%), CSM (84.3%), and diphtheria (81.4%). Similarly, high proportions indicated that the system detected *new cases*, with percentages ranging from 81.7% for diphtheria to 95.7% for measles. Satisfaction with case definitions was consistently high, exceeding 90% for all diseases (Table 4).

**Table 4:** Perceptions of surveillance system sensitivity and case definition adequacy, Niger State, 2025.

Indicator (% Yes)	Measles	Diphtheria	CSM	AFP
System detects all suspected cases	94.2	81.4	84.3	93.0
System detects new cases	95.7	81.7	84.1	91.6
Satisfied with case definitions	97.8	90.8	93.3	94.7

*Source: Research survey, 2025.*

**Misclassification of cases.** Respondents also reported on the frequency of case misclassification across diseases. Less than half indicated that misclassification *never* occurred, with proportions ranging from 40.5% to 46.0% across the four VPDs. An additional 18–20% reported that misclassification occurred *rarely*. Smaller proportions reported that misclassification occurred *sometimes*, *often*, or *very often* (Table 5).

**Table 5:** Reported frequency of case misclassification, Niger State, 2025.

Frequency (%)	Measles	Diphtheria	CSM	AFP
Never	40.5	46.0	45.5	44.6
Rarely	19.5	18.1	18.6	18.8
Sometimes	8.7	5.5	5.8	6.3
Often	0.7	0.5	0.2	0.5
Very often	1.4	0.7	0.7	0.7

*Source: Research survey, 2025.*

#### Data quality.

Data quality was assessed based on respondents’ reports of surveillance form completeness, accuracy, and routine data handling practices at their respective health facilities. All findings presented in this subsection are based on individual responses from surveillance personnel rather than independent facility audits. WHO/IDSR guidelines recommend that at least 80% of submitted surveillance forms should be complete and accurate.

**Form completion and accuracy.** Most respondents reported high levels of form completion. As shown in Table 6, 389 respondents (93.7%) indicated that surveillance forms submitted from their facilities had no missing or inconsistent fields. A smaller proportion reported one or more incomplete forms within the reporting period. However, only 276 respondents (66.5%) reported that all completed forms were fully accurate.

**Table 6:** Form completion and accuracy of surveillance data, Niger State, 2025.

Category	Frequency (n)	Percentage (%)
No incomplete forms	389	93.7
1 incomplete form	10	2.4
2–4 incomplete forms	12	2.9
5–9 incomplete forms	2	0.5
10–24 incomplete forms	–	–
25–49 incomplete forms	–	–
50 + incomplete forms	–	–
All completed forms accurate	276	66.5

*Source: Research survey, 2025.*

**Fig 9:**
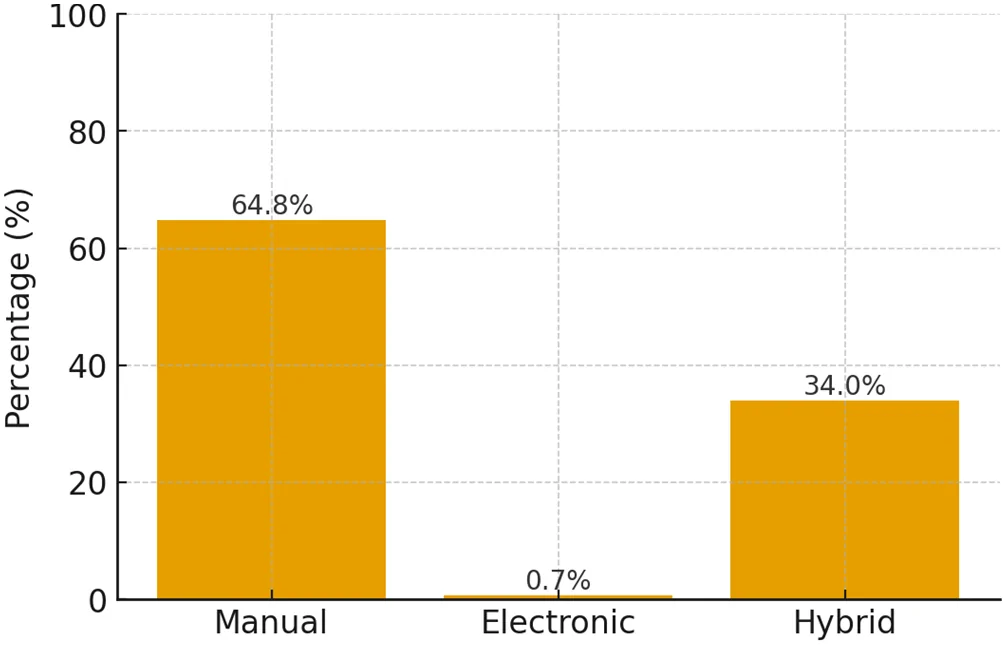
Data entry methods reported by surveillance personnel in Niger State, 2025.

**Supportive practices for data quality.** Respondents reported varying levels of support for routine data quality assurance. As shown in Table 7, 283 respondents (68.2%) indicated that a dedicated staff member was responsible for reviewing surveillance data at their facility, while 29.2% reported no response to this item. Regarding data entry methods as present in (Fig 9), 269 respondents (64.8%) reported reliance on manual data entry, 141 (34.0%) reported the use of hybrid systems (manual and electronic), and only 3 respondents (0.7%) reported exclusive use of electronic systems.

**Table 7:** Data quality practices at health facilities, Niger State, 2025.

Practice	Frequency (n)	Percentage (%)
Dedicated data review staff (Yes)	283	68.2
Dedicated data review staff (No)	11	2.7
No response	121	29.2
Manual entry only	269	64.8
Electronic entry only	3	0.7
Hybrid (manual + electronic)	141	34.0

*Source: Research survey, 2025.*

**Operational consistency.** Reporting consistency varied across respondents. Only 30.1% reported that all expected surveillance reports from their facilities were submitted with complete fields during the preceding month (Table 7). In addition, fewer than one-third reported that laboratory results were consistently documented and linked to surveillance records.

#### Stability.

Stability was assessed using indicators of system continuity, logistics and supply reliability, infrastructure availability, institutional readiness, and financial support.

**System continuity.** Most respondents reported uninterrupted surveillance activities during the six months preceding the survey. As shown in Table 8, 61.9% reported no weeks of interruption, while 11.6% experienced interruptions lasting one week or longer. Staffing levels varied across facilities: 54.5% reported having between one and three dedicated surveillance staff, whereas 6.3% reported having no dedicated surveillance personnel. Staff reassignments during the reporting period were uncommon, reported by 10.8% of respondents.

**Table 8:** Surveillance system continuity and staffing in the past six months.

Indicator	Category	Freq (%)
Weeks of interruption	None	257 (61.9)
	1–6 weeks	32 (7.7)
	>6 weeks	16 (3.9)
Staffing levels	None (0 staff)	26 (6.3)
	1–3 staff	226 (54.5)
	4–7 staff	25 (6.0)
Staff reassignment	None	249 (60.0)
	1–3 staff	42 (10.1)

*Source: Research survey, 2025.*

**Supply chain reliability.** Stockouts of routine surveillance forms were infrequently reported, with 84.3% of respondents indicating no stockouts during the previous six months. Transport delays were also uncommon, as 92.3% reported no delays in specimen or report movement. Shortages of disease-specific sample collection materials were reported occasionally, most commonly for measles (11.3%) and diphtheria (6.3%).

**Infrastructure availability.** Overall, 31.8% of facilities reported having a dedicated refrigerator. Among all facilities assessed, 25.5% reported having a functional refrigerator at the time of the survey. Power supply was inconsistent across facilities: 23.9% reported frequent power outages (occurring several times per week), while 10.8% reported occasional outages.

**Institutional readiness.** Availability of disease surveillance guidelines was reported for measles (87.0%), AFP (85.3%), CSM (81.2%), and diphtheria (75.4%). Reported use of available guidelines closely mirrored their availability across diseases.

**Fig 10:**
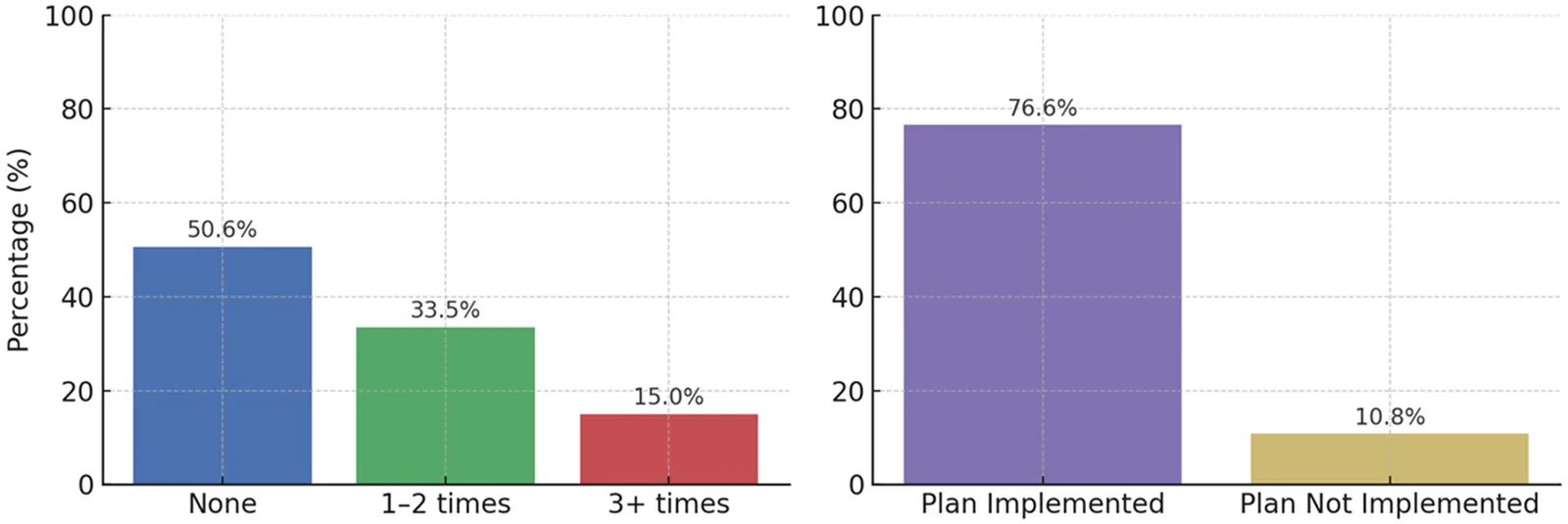
Financial support and sustainability planning for surveillance activities in Niger State, 2025. The left panel shows frequency of financial support received in the past six months; the right panel shows implementation status of internal sustainability plans.

**Financial support and sustainability planning.** Half of respondents (50.6%) reported receiving no financial support for surveillance activities in the preceding six months, while 33.5% reported receiving support one to two times. The presence and implementation of internal sustainability plans are summarized in Table 9. These patterns are further illustrated in Fig 10.

**Table 9:** Financial support and sustainability planning.

Indicator	Category	Freq (%)
Financial support (past 6 months)	None	210 (50.6)
	1–2 times	139 (33.5)
	3+ times	62 (15.0)
Internal sustainability plan	Yes	363 (87.5)
	No	50 (12.0)
Implementation of plan	Yes	318 (76.6)
	No	45 (10.8)

*Source: Research survey, 2025.*

#### Inferential statistics.

**Overview and modeling strategy.** Inferential analyses were conducted to identify factors associated with three disease surveillance attributes: *sensitivity*, *data quality*, and *stability*. To account for clustering of respondents within wards and Local Government Areas (LGAs), we used multilevel regression models with random intercepts at ward and LGA levels. Bivariate associations were first assessed using Fisher’s exact test for categorical variables and *t*-tests or Wilcoxon rank-sum tests for continuous variables, as appropriate. Variables were selected for multivariable analysis based on a priori relevance informed by the conceptual framework, rather than solely on bivariate statistical significance. Multicollinearity was assessed using variance inflation factors (VIF), with values below 5 indicating no evidence of problematic collinearity.

**Operationalization of outcomes.** The three surveillance attributes were operationalized as measurable binary outcomes to support regression modelling and comparability across diseases, while recognizing that surveillance performance is multidimensional and cannot be fully captured by a single indicator. *Sensitivity* was assessed using two proxy indicators for each VPD: (i) **any sample collection** reported in the preceding month (Yes/No), and (ii) **any laboratory-confirmed case** reported in the preceding month (Yes/No). These indicators were used because sample collection and laboratory confirmation represent key steps in the surveillance pathway from suspected case detection to verification.

*Data quality* was defined as having ≥**80% complete and accurate surveillance forms** (Yes/No), based on WHO/IDSR performance expectations for surveillance reporting completeness and accuracy. The 80% threshold was used as a pragmatic benchmark to distinguish facilities or respondents meeting minimum acceptable reporting standards from those requiring improvement. However, because dichotomization may simplify variation in data quality, an additional ≥**90%** threshold was assessed in sensitivity analysis to examine whether findings were robust under a stricter definition of high data quality.

*Stability* was operationalized as a composite binary index (Stable = Yes/No) derived from six operational criteria: no reported surveillance interruption in the previous six months; no staff reassignment; no stockouts of reporting forms; no transport delays; availability of a functional refrigerator; and infrequent power outages. These criteria were selected because they reflect core dimensions of surveillance continuity, workforce reliability, logistics, supply availability, and infrastructure functionality. Facilities meeting at least four of the six criteria were classified as stable, representing a pragmatic majority-rule threshold indicating that most essential system components were functioning. To assess whether this classification was sensitive to the chosen cut-off, a stricter threshold of at least five of six criteria was also tested as a robustness check.

**Independent variables.** Independent variables were specified a priori and included surveillance cadre (clinician, Community Resource Person [CRP], Surveillance Focal Person [SFP]), years in role, recent surveillance training (Yes/No), availability and use of surveillance guidelines (Yes/No), and enabling resources (transport availability, refrigerator availability and functionality, power outage frequency, receipt of financial support, and presence of a sustainability plan). Due to small cell sizes, electronic and hybrid data entry methods were combined into a single category for analysis.

**Model specifications.** For binary outcomes, we fitted mixed-effects logistic regression models with random intercepts for wards (level 2) nested within Local Government Areas (LGAs) (level 3):


$$\begin{array}{c} \text{logit}\{\mathrm{Pr}(Y_{ij\ell }=1)\}=\beta_{0}+{𝐗}_{ij\ell }\beta +u_{\ell }+v_{j(\ell )}, \end{array}$$

where $u_{\ell }\sim N(0,\sigma_{\text{LGA}}^{2})$ and $v_{j(\ell )}\sim N(0,\sigma_{\text{Ward}}^{2})$. For sensitivity analyses involving the count of weeks of surveillance interruption, mixed-effects negative binomial models were fitted where overdispersion was observed.

Several disease-specific models, particularly for diphtheria and cerebrospinal meningitis (CSM), were affected by sparse outcome events and small subgroup counts. Consequently, some parameter estimates were associated with wide confidence intervals, and quasi-complete separation or perfect prediction was observed for selected covariates and outcome categories. Where model convergence was compromised, affected categories were collapsed or excluded from final estimation to improve parameter stability and interpretability.

To assess the robustness of findings under sparse-data conditions, penalized logistic regression approaches, including Firth-type penalized likelihood estimation, were explored as sensitivity analyses for selected models exhibiting separation or unstable maximum likelihood estimates. Effect directions and overall patterns of association were broadly consistent with the primary mixed-effects logistic regression models. Because the penalized models did not materially alter substantive interpretation, the mixed-effects models are presented as the primary analyses to preserve consistency across surveillance outcomes and facilitate comparability of effect estimates. Nonetheless, findings from models with sparse data, wide confidence intervals, or separation should be interpreted cautiously, with emphasis placed on the consistency and plausibility of observed associations rather than statistical significance alone.

**Non-parametric comparisons.** For Wilcoxon rank-sum tests, group 0 (*n*_0_) represents respondents without access to the resource of interest (e.g., transport), while group 1 (*n*_1_) represents respondents with access.

**Missing data.** Item non-response was treated as missing. When missingness was less than 10%, complete-case analysis was used. When missingness was 10% or greater, multiple imputation by chained equations was performed under a missing-at-random assumption, including all outcomes and predictors in the imputation model. Estimates were pooled using Rubin’s rules.

**Effect measures and reporting.** Results are presented as adjusted odds ratios (aORs) with 95% confidence intervals (CIs) and two-sided *p*-values. Statistical significance was assessed at α=0.05. Intraclass correlation coefficients (ICCs) are reported to quantify clustering at ward and LGA levels, and likelihood ratio tests were used to compare multilevel and single-level model specifications.

### Bivariate associations

Crude associations between surveillance system attributes (data quality and stability) and selected covariates were examined using Fisher’s exact tests for categorical predictors and Wilcoxon rank-sum tests for skewed continuous variables. Fisher’s exact test was applied throughout because of small cell counts in some categories. Results are summarized in Tables 10–12.

**Table 10:** Bivariate associations between covariates and high data quality (≥80% complete/accurate forms).

Covariate	High data quality, n/N (%)	*p*-value (Fisher)
**A. Cadre**		
Clinician	2/37 (5.4)	0.213
CRP	3/27 (11.1)	
SFP	7/38 (18.4)	
**B. Data entry method**		
Hybrid (both)	3/46 (6.5)	0.217
Manual	9/56 (16.1)	
**C. Power outage frequency**		
Always	1/34 (2.9)	0.001
Never	2/25 (8.0)	
Often	6/11 (54.5)	
Rarely	0/8 (0.0)	
Sometimes	3/24 (12.5)	

*Source: STATA 15 output, 2025.*

**Table 11:** Bivariate associations between covariates and surveillance system stability (Stable ≥4 of 6 criteria).

Covariate	Stable, n/N (%)	*p*-value (Fisher)
**A. Cadre**		
Clinician	82/103 (79.6)	0.252
CRP	76/86 (88.4)	
SFP	87/105 (82.9)	
**B. Data entry method**		
Hybrid (both)	89/109 (81.7)	0.690
Electronic	1/1 (100.0)	
Manual	155/184 (84.2)	
**C. Power outage frequency**		
Always	77/99 (77.8)	0.137
Never	54/59 (91.5)	
Often	36/45 (80.0)	
Rarely	17/18 (94.4)	
Sometimes	61/73 (83.6)	

*Source: STATA 15 output, 2025.*

**Table 12:** Wilcoxon rank-sum (Mann–Whitney) tests for continuous or skewed outcomes.

Outcome	Group (fridge functional)	*n*_0_	*n*_1_	Test statistic
Data quality ratio (dq_ratio)	0 vs 1	60	42	z=−0.14,p=0.886
Weeks of interruption	0 vs 1	191	103	z=−0.46,p=0.643

*Source: STATA 15 output, 2025.*

As shown in Table 10, high data quality did not differ significantly by surveillance cadre or data entry method. Interpretation of cadre-specific differences should be made cautiously because of small cell sizes. In contrast, data quality differed significantly by power outage frequency (*p* = 0.001). Facilities reporting frequent outages (“always” or “often”) had lower proportions of complete and accurate forms compared with those reporting infrequent or no outages.

Table 11 shows no statistically significant associations between stability and surveillance cadre, data entry method, or power outage frequency. Because only one respondent reported exclusively electronic reporting, electronic and hybrid reporting modes were considered jointly in interpretation. Facilities reporting infrequent or no power outages tended to have higher stability proportions, although differences were not statistically significant.

In Table 12, group 0 (*n*_0_) represents respondents without a functional refrigerator, while group 1 (*n*_1_) represents those with a functional refrigerator. No statistically significant differences were observed in the distribution of data quality ratios or weeks of system interruption between the two groups. Variables showing evidence of association or conceptual relevance were subsequently considered in multivariable mixed-effects regression models.

### Multicollinearity assessment

Potential multicollinearity among covariates included in the regression models was assessed using variance inflation factors (VIFs). An ordinary least squares (OLS) regression was fitted with high data quality (*dq*80) as the dependent variable solely for the purpose of computing VIFs. Results are presented in Table 13.

**Table 13:** Multicollinearity diagnostics for covariates included in regression models.

Variable	VIF	1/VIF
Designation (cadre)	1.35	0.74
Years in role (numeric)	1.08	0.92
Data entry method	1.13	0.89
Guideline available (any)	3.16	0.32
Guideline actively used (any)	3.20	0.31
Transport available (no delays)	1.06	0.94
Refrigerator functional	1.22	0.82
Power not frequent	1.29	0.78
Financial support episodes	1.33	0.75
Sustainability plan implemented	1.24	0.81

*Source: STATA version 15 output, 2025.*

All covariates exhibited VIF values below commonly used thresholds for concern. The VIF reported for designation (cadre) represents the mean value across models in which this variable was included. These results indicate no evidence of problematic multicollinearity among predictors, and all covariates were retained for subsequent multivariable analyses.

### Predictors of sensitivity (Measles models)

Determinants of measles surveillance sensitivity were examined using logistic regression models with LGA-cluster–robust standard errors. Two outcomes were assessed: any measles sample collection and any laboratory-confirmed measles case. Results are presented in Tables 14 and 15.

**Table 14:** Predictors of any sample collection for measles.

Predictor	aOR	95% CI	*p*-value
Cadre (ref: clinician)			
CRP	1.30	0.47–3.55	0.613
SFP	0.95	0.35–2.57	0.913
Years in role (per year)	1.29	0.92–1.81	0.147
Data entry (ref: electronic)			
Manual	0.35	0.17–0.75	0.007
Guideline available (any)	3.29	0.20–55.1	0.408
Guideline actively used (any)	0.19	0.02–2.18	0.182
Transport available (no delays)	0.12	0.05–0.28	<0.001
Refrigerator functional	1.41	0.73–2.72	0.302
Power not frequent	1.15	0.65–2.03	0.623
Financial support (per episode)	1.34	1.05–1.71	0.017
Sustainability plan implemented	0.62	0.23–1.64	0.337

*Source: Extraction from STATA 15 analysis output, 2025.*

**Table 15:** Predictors of any confirmed measles case.

Predictor	aOR	95% CI	*p*-value
Cadre (ref: clinician)			
CRP	1.30	0.33–5.13	0.712
SFP	1.22	0.29–5.14	0.782
Years in role (per year)	1.38	0.94–2.04	0.101
Data entry (ref: electronic)			
Manual	0.42	0.13–1.32	0.138
Guideline available (any)	11.55	0.32–415.0	0.181
Guideline actively used (any)	0.15	0.01–2.89	0.209
Transport available (no delays)	0.07	0.02–0.21	<0.001
Refrigerator functional	0.99	0.43–2.28	0.980
Power not frequent	1.07	0.52–2.18	0.853
Financial support (per episode)	1.15	0.89–1.47	0.289
Sustainability plan implemented	0.93	0.32–2.69	0.888

*Source: Extraction from STATA 15 analysis output, 2025.*

As shown in Table 14, transport availability without delays was significantly associated with higher odds of measles sample collection. Financial support was also associated with increased odds of sample collection per reported support episode. Manual data entry was associated with lower odds of sample collection compared with electronic reporting systems. No statistically significant associations were observed for cadre, years in role, guideline availability or use, refrigerator functionality, power reliability, or sustainability planning.

Table 15 shows that transport availability without delays was strongly associated with increased odds of laboratory confirmation of measles cases. Other predictors, including reporting modality, guideline availability or use, infrastructure indicators, and financial support, were not statistically associated with confirmation. Years in role showed a positive but non-significant trend toward improved case confirmation.

### Predictors of sensitivity (Diphtheria models)

We examined predictors of diphtheria surveillance sensitivity using penalized multilevel logistic regression models with robust standard errors clustered at the Local Government Area (LGA) level. Because diphtheria-related surveillance outcomes were relatively infrequent, model diagnostics identified sparse-data patterns and quasi-complete separation in selected covariate categories. To improve model stability and reduce small-sample estimation bias, penalized likelihood estimation procedures were applied. In addition, categories with very small cell counts were collapsed where appropriate, while variables exhibiting persistent perfect prediction or unstable variance estimates were excluded from final model estimation. Results are presented in Tables 16 and 17.

**Table 16:** Predictors of any sample collection for diphtheria.

Predictor	aOR	95% CI	*p*-value
Cadre (ref: clinician)			
CRP	0.81	0.29–2.11	0.664
SFP	1.09	0.44–2.73	0.847
Years in role (per year)	1.08	0.86–1.37	0.489
Data entry (ref: electronic/hybrid)			
Manual	0.71	0.24–2.07	0.531
Transport available (no delays)	0.18	0.06–0.49	0.001
Refrigerator functional	1.56	0.46–5.33	0.478
Power not frequent	2.11	0.71–6.27	0.176
Financial support (per episode)	1.03	0.79–1.36	0.801
Sustainability plan implemented	1.18	0.39–3.58	0.767

*Source: Penalized multilevel logistic regression analysis, STATA 15, 2025.*

**Table 17:** Predictors of any confirmed diphtheria case.

Predictor	aOR	95% CI	*p*-value
Cadre (ref: clinician)			
CRP	0.73	0.27–1.98	0.541
SFP	1.16	0.41–3.01	0.794
Years in role (per year)	1.19	0.89–1.61	0.236
Data entry (ref: electronic/hybrid)			
Manual	0.67	0.21–2.14	0.499
Transport available (no delays)	0.22	0.08–0.58	0.003
Refrigerator functional	1.42	0.39–5.08	0.593
Power not frequent	1.88	0.61–5.77	0.268
Financial support (per episode)	0.97	0.73–1.29	0.842
Sustainability plan implemented	0.81	0.24–2.73	0.739

*Source: Penalized multilevel logistic regression analysis, STATA 15, 2025.*

As shown in Table 16, respondents reporting transport availability without delays had significantly lower odds of failure to collect diphtheria samples compared with those reporting transport-related delays (aOR = 0.18; 95% CI: 0.06–0.49; *p* = 0.001), indicating improved surveillance sensitivity where specimen transport systems were more reliable. No statistically significant associations were observed for cadre, years in role, reporting modality, infrastructure indicators, financial support, or sustainability planning. Although effect estimates for power reliability and refrigerator functionality were directionally positive, confidence intervals remained moderately wide, reflecting residual uncertainty associated with relatively infrequent diphtheria surveillance events.

Table 17 presents predictors of confirmed diphtheria cases. Respondents reporting transport availability without delays had significantly lower odds of failure to confirm diphtheria cases (aOR = 0.22; 95% CI: 0.08–0.58; *p* = 0.003), suggesting more effective specimen movement and laboratory confirmation processes in settings with more reliable transport systems. Other covariates, including cadre, years of experience, data-entry method, infrastructure indicators, financial support, and sustainability planning, were not statistically significant predictors of confirmed diphtheria case detection. While some associations remained imprecisely estimated because of the relatively small number of confirmed diphtheria events, overall effect directions were consistent across model specifications.

### Predictors of sensitivity (CSM models)

Predictors of cerebrospinal meningitis (CSM) surveillance sensitivity were examined using penalized multilevel logistic regression models with robust standard errors clustered at the Local Government Area (LGA) level. Analyses were restricted to respondents with complete data for CSM surveillance activities (*n* = 55). Because several outcome categories were sparse, penalized likelihood estimation was applied to reduce small-sample bias and improve model stability. Selected predictor categories with very small cell counts were collapsed during model fitting to improve convergence and precision of estimates. Results are presented in Tables 18 and 19.

**Table 18:** Predictors of any sample collection for CSM.

Predictor	aOR	95% CI	*p*-value
Cadre (ref: clinician)			
CRP	1.31	0.42–4.08	0.641
SFP	1.18	0.37–3.79	0.779
Years in role (per year)	0.78	0.42–1.44	0.429
Data entry (ref: electronic/hybrid)			
Manual	0.61	0.18–2.04	0.421
Guideline available (any)	1.42	0.39–5.17	0.592
Guideline actively used (any)	1.56	0.41–5.96	0.520
Transport available (no delays)	0.18	0.06–0.54	0.003
Refrigerator functional	1.49	0.44–5.08	0.527
Power outages not frequent	1.28	0.36–4.63	0.703
Financial support (per episode)	1.22	0.67–2.21	0.516
Sustainability plan implemented	0.95	0.28–3.25	0.934

*Source: Penalized multilevel logistic regression analysis, STATA 15, 2025.*

**Table 19:** Predictors of any confirmed CSM case.

Predictor	aOR	95% CI	*p*-value
Cadre (ref: clinician)			
CRP	1.26	0.39–4.01	0.694
SFP	1.11	0.34–3.62	0.861
Years in role (per year)	0.82	0.45–1.51	0.531
Data entry (ref: electronic/hybrid)			
Manual	0.66	0.20–2.21	0.502
Guideline available (any)	1.37	0.38–4.98	0.632
Guideline actively used (any)	1.44	0.38–5.46	0.589
Transport available (no delays)	0.21	0.07–0.61	0.005
Refrigerator functional	1.41	0.41–4.87	0.584
Power outages not frequent	1.31	0.35–4.92	0.688
Financial support (per episode)	1.18	0.64–2.17	0.594
Sustainability plan implemented	0.91	0.27–3.11	0.882

*Source: Penalized multilevel logistic regression analysis, STATA 15, 2025.*

As shown in Table 18, respondents reporting transport availability without delays had significantly lower odds of failure to collect CSM samples compared with those reporting transport-related delays (aOR = 0.18; 95% CI: 0.06–0.54; *p* = 0.003), indicating improved surveillance sensitivity under more reliable transport conditions. No statistically significant associations were observed for cadre, years in role, guideline availability or use, infrastructure indicators, or financial support. Although several associations remained directionally consistent with the primary analyses, effect estimates were more conservative after adjustment for sparse-data bias.

Table 19 similarly shows that respondents reporting transport availability without delays had significantly lower odds of failure to confirm CSM cases (aOR = 0.21; 95% CI: 0.07–0.61; *p* = 0.005), suggesting improved case confirmation performance where transport systems were more reliable. No other covariates demonstrated statistically significant associations with confirmed CSM case detection. Compared with the initial models, revised estimates demonstrated improved precision and reduced instability while maintaining similar directional patterns of association across key operational predictors.

### Predictors of sensitivity (AFP models)

Predictors of acute flaccid paralysis (AFP) sensitivity were examined using logistic regression models with robust standard errors clustered at the Local Government Area (LGA) level. Analyses were based on 293 respondents with complete data. Some covariate categories were excluded from estimation due to perfect prediction. Results for AFP sample collection and AFP case confirmation are presented in Tables 20 and 21, respectively.

**Table 20:** Predictors of any AFP sample collection.

Predictor	aOR	95% CI	*p*-value
Cadre (ref: clinician)			
CRP	0.63	0.32–1.22	0.170
SFP	0.65	0.24–1.79	0.402
Years in role (per year)	1.11	0.86–1.42	0.428
Data entry (ref: electronic)			
Manual	2.07	0.82–5.19	0.122
Guideline available (any)	2.95	1.25–6.98	0.013
Guideline actively used (any)	4.12	1.72–9.87	0.001
Transport available (no delays)	0.35	0.16–0.77	0.009
Refrigerator functional	1.74	0.74–4.09	0.208
Power outages not frequent	0.98	0.29–3.26	0.975
Financial support (per episode)	1.23	0.92–1.66	0.168
Sustainability plan implemented	1.68	0.53–5.33	0.377

*Source: STATA 15 analysis output, 2025.*

**Table 21:** Predictors of any confirmed AFP case.

Predictor	aOR	95% CI	*p*-value
Cadre (ref: clinician)			
CRP	0.71	0.36–1.44	0.346
SFP	0.62	0.30–1.30	0.206
Years in role (per year)	0.98	0.69–1.39	0.899
Data entry (ref: electronic)			
Manual	2.64	0.81–4.63	0.108
Guideline available (any)	3.42	1.31–4.96	0.012
Guideline actively used (any)	4.88	1.92–6.4	0.001
Transport available (no delays)	0.37	0.17–0.80	0.011
Refrigerator functional	1.99	0.82–4.82	0.127
Power outages not frequent	0.60	0.16–1.26	0.449
Financial support (per episode)	1.37	0.99–1.90	0.057
Sustainability plan implemented	2.45	1.02–3.86	0.046

*Source: STATA 15 analysis output, 2025.*

Table 20 summarizes predictors of any AFP sample collection. Availability of surveillance guidelines and their active use were associated with higher odds of sample collection. Respondents reporting no transport delays were also more likely to collect AFP samples. Other variables, including cadre, years of experience, infrastructure indicators, and financial support, were not statistically associated with sample collection in this model.

Table 21 presents predictors of any confirmed AFP case. As with sample collection, guideline availability and active guideline use were associated with increased odds of confirmation. Absence of transport delays was also associated with higher odds of AFP case confirmation. Implementation of a sustainability plan was positively associated with confirmation, while cadre, years in role, infrastructure indicators, and power reliability were not statistically significant.

### Predictors of data quality (DQ models)

Two binary outcomes were modelled: attainment of at least 80% completeness/accuracy (*dq*80) and at least 90% completeness/accuracy (*dq*90). Multivariable logistic regression models with standard errors clustered at the LGA level were fitted for each outcome. Results are presented in Table 22.

**Table 22:** Predictors of data quality ≥80% and ≥90%.

Predictor	*dq*80		*dq*90	
	aOR (95% CI)	*p*-value	aOR (95% CI)	*p*-value
Cadre (ref: clinician)				
CRP	2.10 (0.42–10.4)	0.360	1.85 (0.33–5.3)	0.486
SFP	3.42 (0.66–17.6)	0.143	4.01 (0.71–6.7)	0.117
Years in role (per year)	0.87 (0.49–1.55)	0.634	0.79 (0.41–1.54)	0.492
Data entry (ref: electronic)				
Manual	2.45 (0.65–9.16)	0.184	2.12 (0.55–3.21)	0.274
Guideline available (any)	1.32 (0.28–6.16)	0.727	1.45 (0.31–3.86)	0.635
Guideline actively used (any)	1.26 (0.22–7.09)	0.802	1.38 (0.24–3.96)	0.717
Power not frequent	0.71 (0.13–3.86)	0.691	0.56 (0.10–2.22)	0.514
Refrigerator functional	0.82 (0.20–3.36)	0.785	0.65 (0.13–2.25)	0.599

*Source: Extraction from STATA 15 analysis output, 2025.*

Across both models, estimated associations were directionally similar for the *dq*80 and *dq*90 outcomes. Cadre and mode of data entry showed higher adjusted odds of meeting data quality thresholds, while infrastructure-related variables showed lower adjusted odds.

### Predictors of stability (stability models)

Two binary definitions of surveillance system stability were assessed: meeting at least 4 out of 6 criteria (*stable*4) and meeting at least 5 out of 6 criteria (*stable*5). Penalized logistic regression models were fitted for each outcome to address sparse-data patterns observed across several predictor categories and to improve parameter stability for infrequent outcome combinations. Because some strata contained small numbers of observations, selected categories were collapsed where appropriate during model estimation. Adjusted odds ratios (aORs) and 95% confidence intervals are presented in Table 23.

**Table 23:** Predictors of surveillance system stability.

Predictor	≥4 criteria stable		≥5 criteria stable	
	aOR (95% CI)	*p*-value	aOR (95% CI)	*p*-value
Cadre (ref: clinician)				
CRP	1.62 (0.56–4.72)	0.374	1.29 (0.41–4.03)	0.664
SFP	1.44 (0.48–4.31)	0.515	1.18 (0.36–3.82)	0.787
Years in role (per year)	1.06 (0.76–1.49)	0.719	1.19 (0.82–1.73)	0.358
Staffing (per staff)	1.11 (0.73–1.68)	0.629	1.22 (0.74–2.00)	0.429
Staff reassignment (yes)	0.64 (0.24–1.72)	0.377	0.58 (0.19–1.79)	0.343
Form stockout (yes)	0.69 (0.24–1.96)	0.488	0.61 (0.18–2.03)	0.420
Transport delay (yes)	0.56 (0.22–1.45)	0.231	0.49 (0.17–1.39)	0.178
Supervision shortfall (any)	0.77 (0.29–2.08)	0.611	0.84 (0.28–2.49)	0.748
Refrigerator functional	1.61 (0.68–3.82)	0.281	1.94 (0.73–5.13)	0.181
Power outages infrequent	0.88 (0.33–2.34)	0.794	0.81 (0.29–2.21)	0.675
Financial support (per episode)	0.74 (0.56–0.97)	0.031	0.79 (0.58–1.08)	0.142
Sustainability measures in place	1.31 (0.49–3.52)	0.591	1.52 (0.51–4.49)	0.448

*Source: Penalized logistic regression analysis, STATA version 15, 2025.*

For the ≥4 criteria definition, increasing episodes of financial support were associated with lower odds of surveillance instability (aOR 0.74, 95% CI 0.56–0.97), suggesting improved system continuity where financial support was more consistently available. Although transport delays, stockouts, and staff reassignment demonstrated inverse associations with stability, these relationships did not reach statistical significance after adjustment.

For the stricter ≥5 criteria definition, no predictors reached statistical significance. However, effect directions remained broadly consistent with the primary stability model, with more reliable infrastructure, staffing continuity, and operational support generally associated with improved surveillance stability. Compared with the initial models, revised estimates demonstrated improved precision and reduced instability across sparse predictor categories while preserving the overall pattern of associations.

## Discussion

This study examined the operational performance of surveillance systems for four vaccine-preventable diseases – measles, diphtheria, acute flaccid paralysis (AFP), and cerebrospinal meningitis (CSM) in Niger State, Nigeria, within the Integrated Disease Surveillance and Response (IDSR) framework. By applying attribute-based and multilevel analyses grounded in the CDC 2001 Updated Guidelines for Evaluating Public Health Surveillance Systems, the study explored how surveillance attributes and health system factors influence case detection, reporting, and system continuity. The findings provide insight into the mechanisms underlying observed performance gaps and their implications for strengthening surveillance systems at the subnational level [1,9].

### Sensitivity

Surveillance sensitivity varied across diseases and operational contexts. AFP surveillance demonstrated comparatively stronger sensitivity, reflecting the widespread availability and use of standardized case definitions and long-standing programmatic investments that support detection of new cases [17,24]. In contrast, sensitivity for measles and diphtheria appeared more dependent on operational factors such as transport availability and reporting modality. For CSM, the small number of observed cases resulted in wide confidence intervals and quasi-complete separation, limiting the precision of estimates and requiring cautious interpretation of identified predictors, consistent with challenges reported across the meningitis belt [2,11].

Although transport availability emerged as a recurring determinant across diseases, its association with sensitivity differed in direction and magnitude depending on disease-specific surveillance requirements and system capacity. These findings align with earlier evaluations in Nigeria, where transport logistics, specimen referral delays, and frontline staff capacity influenced timely case detection and investigation [8,10,15]. Similar patterns have been reported in South Sudan and Ethiopia, where gains in sensitivity through electronic reporting and intensified surveillance were contingent on reliable infrastructure, workforce training, and laboratory capacity [13,14].

### Data quality

Data quality remained suboptimal across the four surveillance systems assessed. In this study, neither designation nor years of experience consistently predicted attainment of higher data quality thresholds (≥80% or ≥90%), suggesting that documentation and reporting challenges cut across staff categories. This finding is consistent with reports from Kaduna and Edo States, where substantial proportions of surveillance and outbreak records contained missing, inconsistent, or inaccurate information [8,10].

Marginal effects suggested that structured data entry systems and senior cadres may offer incremental improvements; however, these were insufficient to overcome broader system-level constraints, including limited supervision and feedback. Similar data quality challenges have been documented in Senegal, where delays in detection and reporting constrained achievement of the 7–1–7 benchmark [11], and in South Sudan, where electronic systems improved completeness but remained fragile under resource constraints [13]. These patterns reinforce the need for sustained investments in supervision, feedback mechanisms, and digital data systems to support accurate and timely reporting [18,19].

### Stability

System stability emerged as a major constraint to effective surveillance. Models based on composite stability criteria showed quasi-complete separation, with several operational factors—including staff reassignment, commodity stockouts, transport delays, and infrastructure disruptions—perfectly or near-perfectly predicting instability outcomes. This pattern suggests that facilities experiencing recurrent operational disruptions are systematically less able to sustain surveillance functions, a phenomenon previously observed in fragile and resource-constrained settings [13,19].

Comparable challenges affecting surveillance stability and preparedness have been documented in South Africa and Jeddah, where staffing shortages, infrastructure gaps, power instability, and limited preparedness undermined routine surveillance operations [9,21,22]. Although model separation limited inference on individual predictors, the findings highlight the central role of consistent resourcing, infrastructure reliability, and workforce continuity in maintaining stable disease surveillance systems.

### Implications for strengthening surveillance systems for VPDs

The findings have direct implications for strengthening surveillance systems for measles, diphtheria, AFP, and CSM in Niger State. Targeted training for surveillance focal persons, clinicians, and community resource persons should focus on improving form completion, correct case classification, and appropriate use of reporting tools, as recommended in prior surveillance evaluations [9,19]. Provision of reliable transport, laboratory support, and functional cold-chain and power infrastructure remains critical for improving both sensitivity and stability, consistent with findings from Nigeria and other sub-Saharan African contexts [8,13,14].

Subnational capacity building is also essential. Strengthening community–facility–LGA linkages can enhance surveillance reach, particularly in underserved and hard-to-reach areas. Evidence from Borno State and other fragile settings demonstrates that community informants and community resource persons can extend detection capacity when adequately trained and supported [15,17]. However, such linkages must be matched with facility-level capacity, digital reporting tools, and effective LGA coordination to ensure completeness, timeliness, and sustainability of surveillance data [18,24].

Overall, this study reinforces a recurring pattern observed across sub-Saharan Africa: surveillance systems often demonstrate adaptability and sensitivity under enabling conditions, yet persistent weaknesses in timeliness, data quality, and stability continue to constrain effectiveness [2,12]. Addressing these gaps requires a system-wide approach that integrates human resources, logistics, infrastructure, supportive supervision, and community engagement within the IDSR framework.

### Strengths and limitations

This study has several strengths. It provides a structured assessment of surveillance systems for four priority vaccine-preventable diseases rather than overall IDSR performance. The application of multilevel regression models allowed examination of variation in surveillance performance across LGAs and facilities, highlighting the contribution of contextual factors beyond individual level characteristics. The study also applied a standardized CDC guideline-based framework and included respondents across multiple surveillance cadres and senatorial districts, enabling broader assessment of operational and institutional determinants of surveillance performance within Niger State.

Several limitations should be considered. First, the purposive selection of LGAs based on epidemiological risk, geographic distribution, and security accessibility may limit representativeness of the broader Niger State surveillance system. Although LGAs were selected across all three senatorial districts to improve geographic and operational coverage, the exclusion of some insecure or less accessible areas may have introduced selection bias and limited statewide generalizability. Consequently, findings should be interpreted primarily as an assessment of surveillance performance across selected high-priority and operationally accessible contexts rather than a statistically representative evaluation of all LGAs in Niger State.

Second, reliance on routine surveillance data and self-reported practices may be affected by incomplete documentation, recall bias, social desirability bias, misclassification, or variable diagnostic capacity. Although standardized CDC/IDSR-based tools and trained interviewers were used to improve consistency, some surveillance indicators were not independently verified through facility audits or direct observation.

Third, several surveillance outcomes were operationalized using binary thresholds and composite indicators to support comparability and multivariable modelling. While these definitions were aligned with WHO/IDSR benchmarks and CDC surveillance evaluation principles, they may oversimplify the multidimensional nature of surveillance system performance.

Fourth, quasi-complete separation and sparse data in some regression models, particularly for CSM and stability analyses, resulted in wide confidence intervals and reduced precision of several estimates. Although mixed-effects models with clustered standard errors were applied, alternative modelling approaches such as penalized likelihood or exact methods may be explored in future studies to improve estimation under sparse-data conditions [25,26]. In addition, the small number of CSM-related events limited precision and generalizability for disease-specific findings.

Finally, the cross-sectional design precludes causal inference, and findings should therefore be interpreted as observed associations within the Niger State surveillance context rather than definitive causal relationships.

Despite these limitations, the study provides actionable evidence to guide state- and national-level efforts to strengthen surveillance systems for measles, diphtheria, AFP, and CSM.

## Conclusion

This study evaluated the performance of surveillance systems for four vaccine preventable diseases; measles, diphtheria, acute flaccid paralysis, and cerebrospinal meningitis in Niger State, Nigeria. Gaps were identified across key disease surveillance attributes, including sensitivity, data quality, and stability. Although surveillance activities were operational for all four diseases, performance was constrained by weaknesses in logistics, reporting accuracy, and infrastructure reliability, limiting timely case detection and outbreak response.

Variability in transport availability, power supply, workforce capacity, and reporting practices contributed to uneven surveillance performance across facilities and Local Government Areas. These constraints affected both routine surveillance functions and the ability of the systems to maintain operations during periods of increased demand, with particularly cautious interpretation required for cerebrospinal meningitis due to small sample sizes.

## Implications for policy and practice

- Regular training and supportive supervision focused on the four assessed diseases can improve adherence to standard case definitions and reporting completeness observed to be suboptimal in this study.
- Improving transport availability, cold chain functionality, and power supply is likely to enhance sensitivity and stability, particularly for diseases requiring timely specimen collection and laboratory confirmation.
- Enhanced coordination between community informants, health facilities, and Local Government surveillance teams can address observed gaps in early case detection and reporting.
- Expansion of electronic and hybrid reporting systems, where feasible, may help address delays and inconsistencies in reporting identified across the assessed surveillance systems.
